# Three-dimensional tooth morphology in patients with tooth agenesis and its association to agenesis pattern, severity, and sex

**DOI:** 10.1038/s41598-025-11034-6

**Published:** 2025-08-01

**Authors:** Chihiro Tanikawa, Miyuki Nakamura, Takashi Yamashiro

**Affiliations:** Department of Orthodontics and Dentofacial Orthopedics, Graduate School of Dentistry, Osaka University, 1-8 Yamadaoka, Suita, Osaka, 565-0871 Japan

**Keywords:** Incisor, Molar, Japan, Models, Dental, Tooth diseases, Cluster analysis, Oral anatomy, Translational research, Dental anthropology, Orthodontics, Dentistry

## Abstract

Non-syndromic tooth agenesis (TA) can affect both the size and shape of teeth and may interact with sex. This study aimed to advance our understanding of tooth developmental mechanisms by constructing a multifactorial model integrating 3D dental morphology, TA patterns, severity, and sex differences. Digital dental models of 255 Japanese individuals (control: 187; TA: 68) were analyzed. We assessed the contributions of size and shape to sex- and TA-related differences in maxillary central incisors (UI) and first molars (UM) using surface distance mapping and a tooth agenesis-associated shape difference (TAShD) analysis. Additionally, permutation tests were conducted to evaluate the relationship between sex- and TA-associated morphological variation. TA patterns were further classified using k-means clustering, and their associations with 3D tooth morphology were examined. Surface distance maps revealed TA-related morphological traits in both UI and UM, including generalized size reduction, altered cingulum morphology, and diminished distolingual cusps. The TAShD analysis indicated that TA and sex independently influenced tooth shape, although both contributed to size variation. Three distinct TA patterns were identified: Cluster 1 (premolar agenesis) exhibited size reduction without significant shape changes; Cluster 2 (incisor and canine agenesis) showed alterations in both the size and shape of the UI; and Cluster 3 (agenesis of canines, premolars, and second molars) presented with size reduction and shape abnormalities, particularly in the UM. These morphological distinctions across clusters reflect the independent effects of TA and sex on the dental morphology. Collectively, these findings highlight the presence of distinct TA patterns and their associated shape characteristics, thereby offering novel insights into the multifactorial nature of tooth development.

**Supplementary Information:**

The online version contains supplementary material available at 10.1038/s41598-025-11034-6.

## Introduction

The shape of teeth arises from spatiotemporal phenotypic expression influenced by both genetic^1–4^ and environmental factors^5,6^ during morphogenesis. Since tooth morphology is largely determined prenatally and undergoes minimal changes after eruption, analyzing these phenotypic traits provides insights into the mechanisms underlying tooth development.

Among the key factors influencing tooth morphology, sex-related differences and non-syndromic tooth agenesis (TA) are particularly well-documented. While sex-related variations result from both genetic and environmental influences^6^, TA is primarily associated with genetic factors^7–9^. For example, twin studies have suggested that prenatal environmental conditions, such as androgen exposure, significantly influence molar morphology^6^, contributing to sexual dimorphism in teeth. In contrast, TA is predominantly inherited in an autosomal dominant manner with high penetrance and considerable phenotypic variability, both within and among families^7–9^.

The relationship between sex-related differences and the TA appears to be interrelated. Epidemiological studies and meta-analyses have consistently shown that TA is more prevalent in females than in males^10–13^. Brook et al.^14^ proposed a multifactorial model with a continuous scale linking TA and tooth size, suggesting that smaller teeth are associated with higher incidences of TA and a female predisposition, while larger teeth are linked to a tendency for hyperdontia and males. However, the extent to which tooth number, size, and shape can be integrated into a single continuous scale, and the influence of sex differences on these interrelationships remains unclear.

Thus, our first hypothesis posited that there are interactions between sex- and TA-related differences in tooth size and shape (Question #1). It is essential to investigate the underlying causes of this relationship, whether these factors are independent or interdependent. One potential explanation may lie in the distinct patterns of tooth agenesis and TA severity identified in previous studies, such as posterior patterns involving the absence of molars and premolars (often linked to MSX1 and PAX9 mutations)^9,15^, anterior patterns affecting the canines and/or incisors, and mixed patterns involving both premolars and lateral incisors^16^. Each pattern is likely influenced by distinct genetic, environmental, and sex-related factors. Furthermore, the severity of TA has been shown to correlate with reductions in tooth size and alterations in tooth shape^17–19^, with an increasing number of missing teeth being associated with smaller crown dimensions^20–22^. Severe cases of TA also exhibit pronounced morphological anomalies in specific teeth such as the mandibular first molar^23^. Consequently, our second hypothesis was that there are associations between TA patterns, severity, and three-dimensional (3D) tooth morphology (shape and size), which may provide further insights into the mechanisms underlying tooth development (Question #2).

Advancements in geometric morphometrics have enabled precise quantification of sexual dimorphism using 3D models. For example, a previous study^24^ applied homologous modeling, in which the facial shape was consistently represented by a high-resolution template mesh, to analyze 3D facial morphology. This approach allowed them to quantify sexual shape dimorphism (SShD) and effectively distinguish size-dependent (allometric) from size-independent (non-allometric) shape differences. Building on this method, we extended its application to tooth morphology and proposed the development of a novel metric, tooth agenesis-associated shape difference (TAShD), using homologous modeling to quantify and differentiate size-dependent and size-independent TA-associated variations in tooth shape. This approach allows us to assess whether TA-related morphological differences arise independently of sex-related factors.

Thus, this study aimed to provide new insights into the mechanisms underlying tooth development by constructing a multifactorial mathematical model incorporating 3D tooth morphology, size, TA patterns, TA severity, and sex differences. We aimed to: 1) quantify size- and shape-related contributions to TA-associated differences using homologous modeling and TAShD for maxillary central incisors (UI) and first molars (UM); 2) investigate the relationship between sexual dimorphism and TA-associated differences (Question #1); and 3) examine the association between agenesis patterns and 3D tooth morphology in TA patients (Question #2).

## Materials and methods

Following extensive dialogue with the Research Ethics Committee, Osaka University Dental Hospital, approval for an opt-out consent method was given. The study received ethical approval for the use of an opt-out methodology based on the low risk to the patient based on unbiased information. In this interpretation consent is an indication of willingness rather than refusal and informed consent is obtained by generally accessible information as well as easy modes to opt out. Information regarding the study was disclosed on the website of Osaka University Dental Hospital, and participants were given the opportunity to opt out. Accordingly, informed consent was obtained from all participants and/or their legal guardians. All procedures were conducted in accordance with relevant guidelines and regulations, and the study protocol was approved by the Research Ethics Committee of Osaka University Dental Hospital (Approval No.: R1-E8).

### Samples

A total of 255 pre-treatment orthodontic Japanese patients were included in the control (n = 187 [male = 92; female = 95]) and tooth agenesis (TA; n = 68 [male = 37; female = 31]) groups. Details of the sample size estimation, as well as the inclusion and exclusion criteria for the control and TA groups, are outlined below:

1. *Sample size* The sample size was estimated based on a previous study^17^ that reported labiolingual diameters of the UI and UM in both female and male participants across TA and control groups. For sex comparisons, the required sample size ranged from 59 to 84 participants, assuming 90% power (β = 0.1) and α = 0.05–0.01. In contrast, comparisons between TA and control groups required smaller sample sizes (11–15 for UI and 9–13 for UM) due to relatively large intergroup differences compared to the standard deviation. Therefore, a control:TA ratio of 2:1 to 3:1 was adopted to determine the TA group size. Accordingly, the control group size was set at approximately 59–84 per sex, and the TA group at 20–42 per sex. Sample size calculations were performed using the power.t.test function in R (version 4.5,1; https://www.r-project.org/).
2. *Inclusion criteria* All Japanese patients aged 6–35 years who visited the Department of Orthodontics at Osaka University Dental Hospital between 2016 and 2019 for orthodontic diagnosis and treatment planning were included. The control group comprised individuals with mild to moderate malocclusion and without severe TA. The TA group included all patients diagnosed with severe congenital TA, defined as the absence of more than six permanent teeth, excluding third molars. The initial inclusion criteria were assessed from 2016 to 2019. However, to balance the number of males and females in the control group, the inclusion period for males was extended to 2014–2019. For the TA group, this period was further extended to 2012–2019 due to the limited number of patients.
3. *Exclusion criteria* Dental models, medical charts, intraoral photographs, and panoramic radiographs from the hospital’s first-visit records were reviewed by one of the authors (MN). Dental models were fabricated from impressions of the entire dentition, obtained using alginate impression material (Aroma Fine Plus Set, GC Corporation, Tokyo, Japan) and cast with hard plaster (New Plastone II White, GC Corporation, Japan). Syndromic patients (e.g., cleft lip or palate), patients with medical or dental history affecting tooth morphology (e.g., cancer treatment and trauma), and patients with caries or restorations in the UI or UM were excluded. Individuals in the control group with any congenitally missing teeth (excluding third molars) were excluded to minimize potential effects of mild to moderate tooth agenesis. Patients with evident gingival swelling or recession were also excluded. When necessary, intraoral photographs and panoramic radiographs were used to confirm the presence of missing teeth, restorations, caries, and gingival conditions. Due to a reduced number of teeth, patients with TA may exhibit smaller occlusal contact areas, leading to increased tooth wear compared to controls. To ensure accurate 3D morphological analysis of dental crowns by minimizing potential confounding effects from post-eruptive alterations, tooth wear of the UI or UM was assessed by one of the authors (MN) using the Tooth Wear Index^25^; teeth with scores greater than 2 were excluded from the analysis. Tooth crowns (UI and UM) with rough surfaces, unerupted teeth, severe crowding (lacking surface), teeth affected by air bubbles, or any defects compromising the accuracy of the analysis were also excluded.

### Sample demographics

**Fig. 1:**
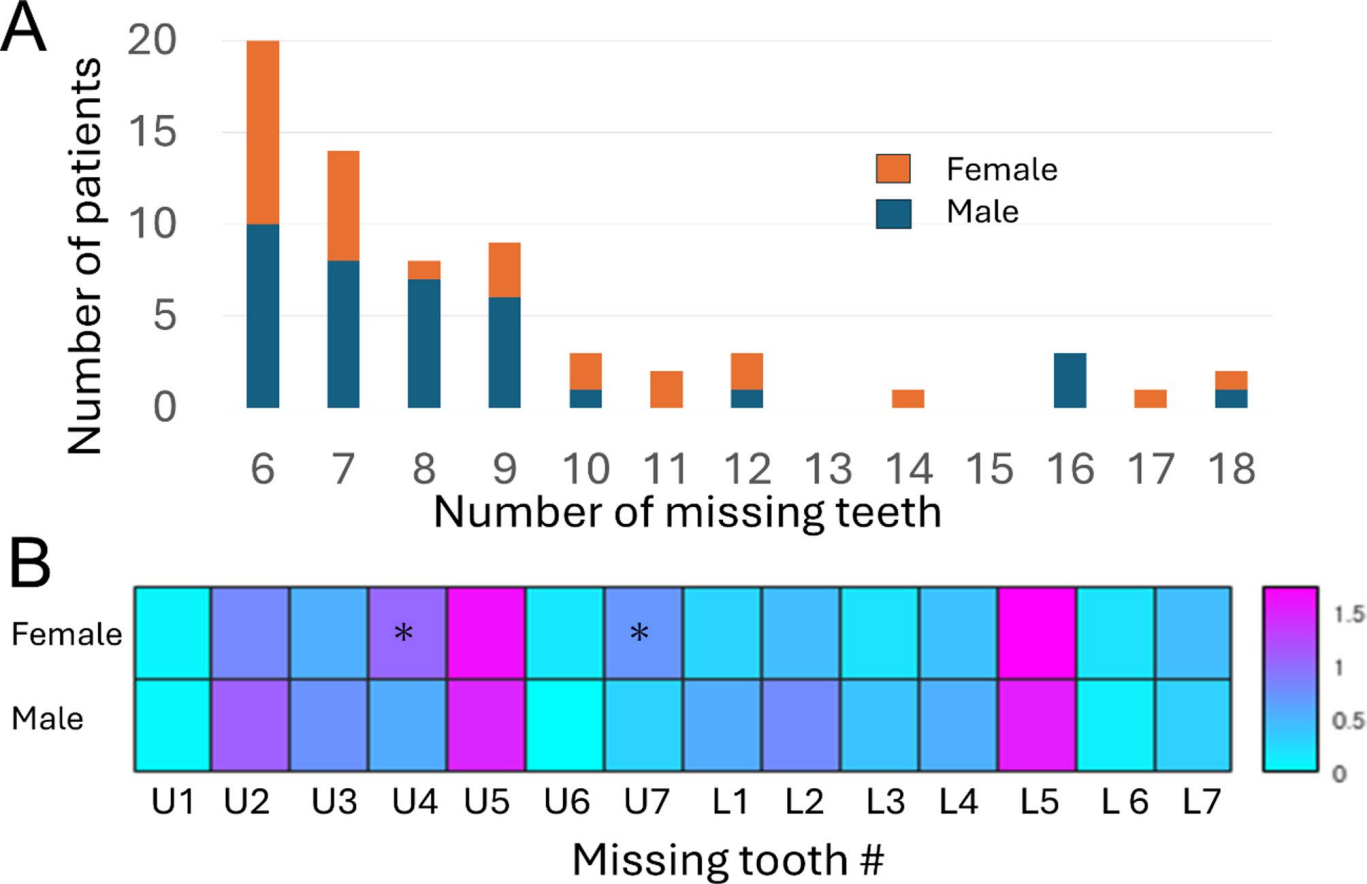
(**A**) Distribution of patients according to the number of missing teeth per subject. (**B**) Distribution of missing tooth locations by sex. The color scale indicates the number of missing teeth at each location.

Finally, the control group, comprising 146 individuals (male = 75; female = 71) for UI and 140 (male = 69; female = 71) for UM, and the TA group, comprising 64 (male = 35; female = 29) and 53 (male = 32; female = 21) individuals for UI and UM, respectively, were included in the analysis (Table 1). Differences in mean age were assessed using independent two-sample t-tests, and sex distributions were compared using chi-square tests. No significant differences were observed (*p* > 0.05), confirming that the groups were demographically matched. The number of patients with congenital missing teeth in the TA group ranged from 6 to 18, as shown in Fig. 1. Females with TA had a significantly higher number of missing maxillary first premolars and second molars (t-test, *p* < 0.05), with most missing teeth found in the maxillary and mandibular second premolars.

**Table 1:** Matched summary of control and tooth agenesis (TA) groups by tooth type, sex, and age with statistical comparisons.

Tooth type	Sex	Control (N, Age ± SD)	TA (N, Age ± SD)	Age *p*-value	Sex distribution *p*-value (Chi-square)
UI	Male	75, 10.5 ± 2.5 y	35, 11.6 ± 3.5 y	0.678 (NS)	0.77 (NS)
	Female	71, 11.0 ± 2.7 y	29, 10.2 ± 3.3 y	0.946 (NS)	
UM	Male	69, 10.6 ± 2.6 y	32, 11.6 ± 3.5 y	0.218 (NS)	0.22 (NS)
	Female	71, 11.2 ± 2.7 y	21, 10.8 ± 3.3 y	0.147 (NS)	

TA, tooth agenesis; UI, upper central incisor; UM, upper first molar; SD, standard deviation; p-value, probability value; y, years; NS, not significant.

Sample sizes and age distributions (mean ± standard deviation) are shown for the control and TA groups, stratified by tooth type and sex.

### UI and UM digital models

Digital dental models were generated under the following conditions: selected plaster models were scanned with 7-μm precision using a 3Shape E3 Dental Scanner (3Shape A/S, Denmark). The 3D surface data of the digital models were exported as stereolithography (STL) files and imported into a measurement software program (HBM-Rugle, version 4.0, Medic Engineering Co., Kyoto, Japan; http://www.rugle.co.jp/hbm/index.html). Crowns of the right UI and right UM were visually extracted at the gingival margin on a 23-inch monitor (Dell U2312HM, Dell, USA) with a maximum preset resolution of 1920 × 1080.

### Coordinate systems and landmark identifications

UI and UM were standardized using a new coordinate axis (Fig. 1A), which was developed in a preliminary study described in Supplementary Text S1, Supplementary Table S1, and Supplementary Figures S1, S2, and S3.

On the 3D surface data of the crown, 16 anatomical landmarks for UI and 17 points for UM (Supplementary Figs. S4 and S5 and Supplementary Tables S1-1 and S2-2) were identified by visual inspection and digitized using a computer mouse cursor and homologous model support software (HBM-Rugle, Medic Engineering Co., Kyoto, Japan). For landmark selection, we conducted preliminary experiments for inter- and intra-observer reliability tests for landmark identification (Supplementary Text S2, Supplementary Tables S3, S4, and S5). Landmarks with an absolute difference of < 2 mm were included in the study. The included landmarks showed a mean absolute difference of 0.32 mm (range: 0.07–0.52 mm) between repeated measurements in both the inter- and intra-observer reliability tests. Additionally, both results fell within the range considered highly reliable based on intra-class correlation coefficients.

### Homologous modeling

**Fig. 2:**
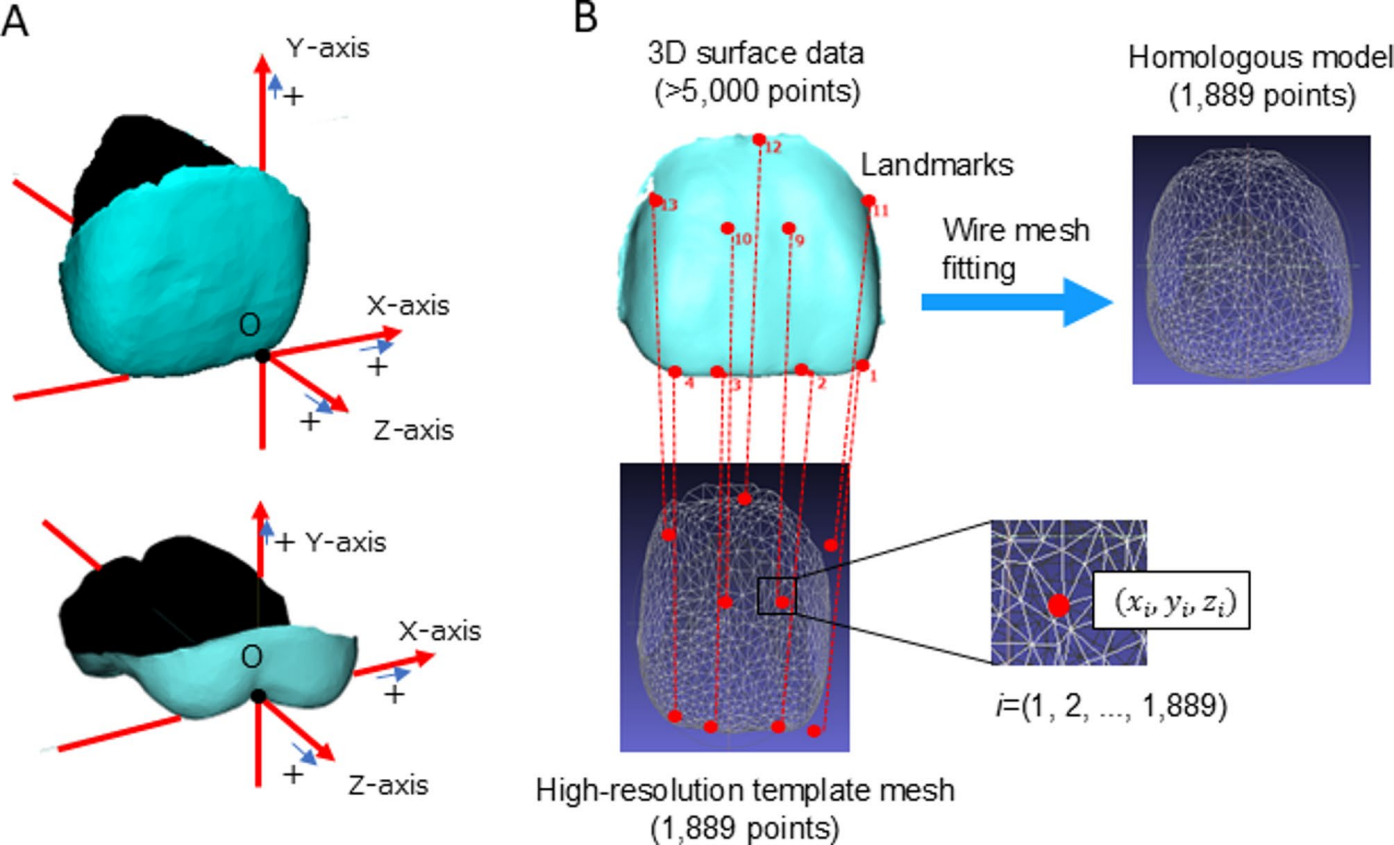
Coordinate system (**A**) and overview of homologous modeling (**B**). (**A**) The origin was defined as the mesial point of the incisal edge of the central incisor and junction of the buccal groove with the occlusal surface of the first molar. The coordinate system was defined such that the X-axis corresponded to the mesiodistal direction, the Y-axis corresponded to the tooth’s axial direction, and the Z-axis corresponded to the buccolingual direction. For definitions, please refer to Supplementary Text S2. (**B**) Based on the anatomical landmarks identified in the 3D surface data, wire mesh fitting was performed on the 3D surface data of the crown of each patient.

An overview of the homologous modeling approach used in this study is presented in Fig. 2. Based on the identified anatomical landmarks, wire mesh fitting was applied to the 3D crown surface data of each patient using the homologous modeling method^26^. A high-resolution template mesh consisting of 1889 points for the UI and 1842 points for the UM was employed. The homologous modeling method aligns the template mesh to the patient’s 3D surface data using an iterative nearest-neighbor algorithm, followed by nonrigid alignment to minimize external and internal energies. External energy is based on the Euclidean distance between the corresponding points, whereas internal energy is derived from mesh deformation. This technique enables the extraction of relevant surface anatomy from tooth data while smoothing or removing irrelevant features, resulting in high-resolution 3D surface data. The final output offers sufficient detail for a quantitative analysis while maintaining small file sizes, making it portable and compatible with various visualization technologies.

### Statistical analysis

Details of the analysis are provided in Supplementary Text S3. Briefly, the analysis comprised four main phases:

1. *Surface displacement.* UI and UM models were standardized via Procrustes analysis (translation, rotation, and centroid size [CS]-based scaling). Averaged 3D tooth shapes were generated for each subgroup (TA/control × sex), and surface displacements between TA and control groups were quantified at 1889 (UI) and 1842 (UM) mesh points along the X-, Y-, and Z-axes. Results were visualized as distance and significance maps.
2. *Dental morphospace.* Principal component analysis (PCA) was performed on the homologous model coordinates of UI and UM. Principal components (PCs) with cumulative contributions exceeding 80% were retained to construct the dental morphospace.
3. *TAShD and allometry.* TAShD was calculated by projecting individual shapes onto the vector connecting the mean shapes of control and TA groups in morphospace. Values below − 1 indicated hyperagenesis, while values above 1 indicated hypernormality^27^. TAShD was further decomposed into allometric and non-allometric components using CS-based regression. Differences between groups (TA vs. control) and between sexes were evaluated using Welch’s t-tests and permutation tests with 1000 iterations.
4. *Tooth agenesis patterns.* Panoramic radiographs were used to identify missing teeth, which were coded as binary vectors by merging left and right data. Agenesis patterns were classified via k-means clustering, with the number of clusters determined using the elbow method. An analysis of variance (ANOVA) was used to assess differences in TAShD components and the number of missing teeth among clusters.

## Results

### Surface displacement in UI

**Fig. 3:**
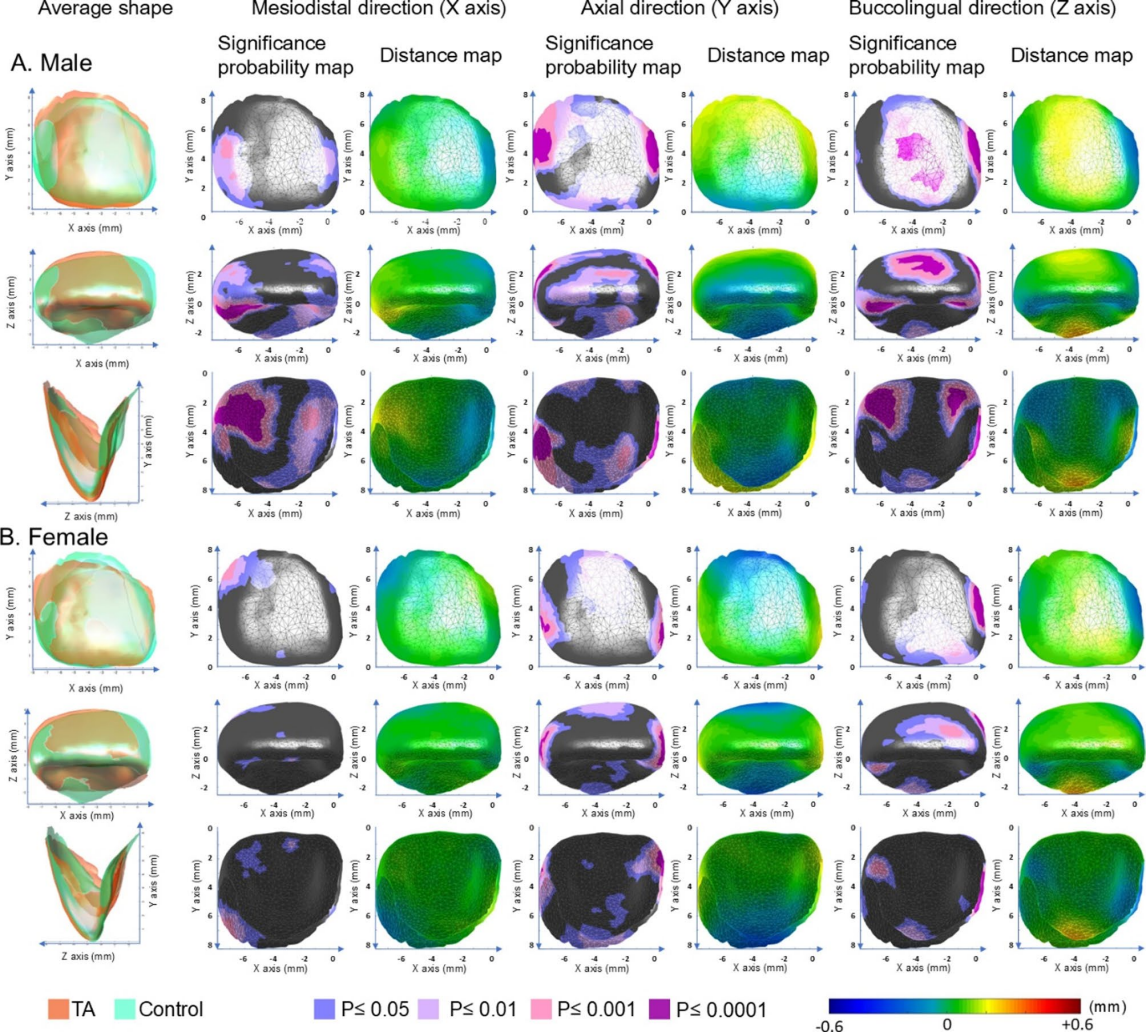
Significance probability maps and distance maps for the maxillary central incisors in the TA and control groups in males (**A**) and females (**B**). The left column represents the average shapes of the TA (red) and control (green) groups. The second and third columns illustrate differences along the mesiodistal (X), axial (Y), and buccolingual (Z) directions, from left to right. In the significance maps, gray indicates non-significant regions, while significant areas are color-coded by *P*-value: blue (*P* < 0.05), pale purple (*P* < 0.01), pale pink (*P* < 0.001), and dark purple (*P* < 0.0001). In the distance maps, color gradients represent TA–control differences. For the mesiodistal direction, yellow indicates a more mesially positioned surface in TA than in control, while blue indicates a more distally positioned surface. For the axial direction, yellow indicates a surface located more apically in the TA group relative to the control group, whereas blue indicates a surface closer to the incisal edge. For the buccolingual direction, yellow indicates a more labially positioned surface in the TA group relative to the control group, whereas blue represents a more lingually positioned surface.

Overall, TA teeth exhibited a narrower and more elongated columnar shape with more prominent marginal ridges and reduced cingulum prominence in comparison to the control group (*p* < 0.05; Table 2, Fig. 3).

**Table 2:** Shape differences between tooth agenesis (TA) and control groups in tooth surface displacement of the maxillary central incisors (UI) and first molars (UM) when excluding size effect.

Tooth	Direction	Findings	Interpretation
UI	Mesiodistal (X)	Male: + X at distal surface and marginal ridge (TA > Control); − X at mesial surface and cingulum (TA < Control) Female: + X at distal cervical surface	Males with TA showed a smaller width-height ratio relative to control. Females with TA showed a reduced mesial cervical width relative to control
	Axial (Y)	Both sexes: + Y at cervical region of labial surface; -Y at incisal tip and cingulum	In both sexes, the cervical region in individuals with TA was positioned closer to the root; the incisal tip and cingulum showed greater height relative to control when excluding size effect
	Buccolingual (Z)	Both sexes: + Z at labial surface and cingulum; − Z at mesial and distal marginal ridge and mesial surface	In both sexes, the labial surface and cingulum in individuals with TA were positioned more labially, and the mesial and distal marginal ridge and mesial surface were positioned more lingually relative to control
UM	Mesiodistal (X)	Both sexes: + X at DL cusps; − X at triangular ridge of DB cusp Female: − X at ML cusp	In both sexes, individuals with TA showed a more mesially positioned DL cusp and a more distally positioned DB triangular ridge. Additionally, females with TA had a more distally positioned ML cusp relative to control females
	Axial (Y)	Both sexes: + Y at DL cusp and mesial pit and mesial marginal ridge; − Y at distal oblique groove and MB cusp Female: + Y at lingual surface of ML cusp	In both sexes, individuals with TA showed reduced DL cusp, mesial pit, and mesial marginal ridge heights, but increased oblique groove and MB cusp heights. Additionally, females with TA exhibited a reduced ML cusp height relative to control females
	Buccolingual (Z)	Both: + Z at DL cusp; − Z at ML cusp and lingual groove Female: + Z at labial surface of DB cusp and buccal groove; − Z at mesial marginal ridge	In both sexes, individuals with TA exhibited a more buccally positioned DL cusp and a more lingually extended ML cusp and lingual groove. Additionally, females with TA showed a buccally positioned DB labial surface and buccal groove, and a more lingually extended mesial marginal ridge relative to control females

**Fig. 4:**
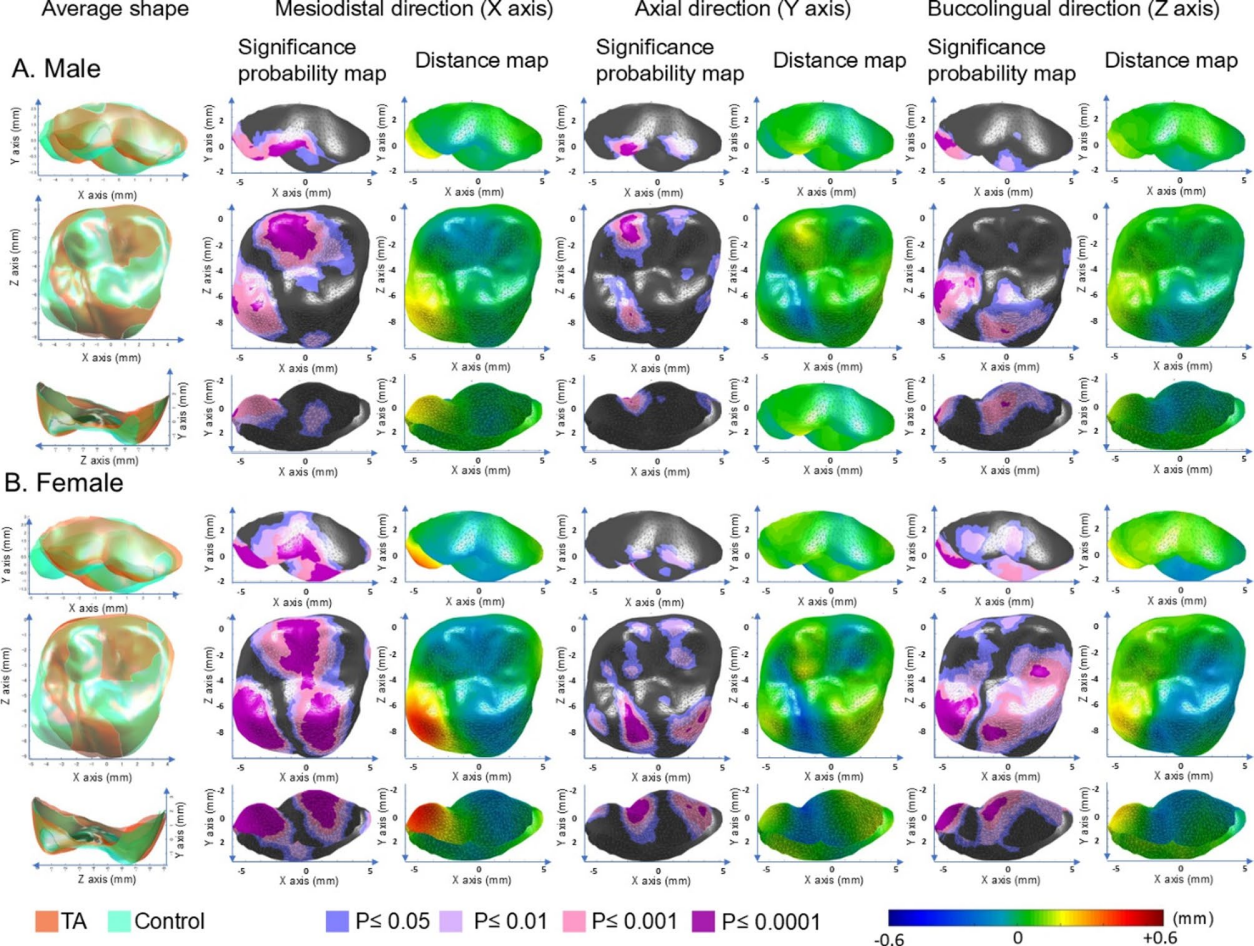
Significance probability maps and distance maps for the maxillary first molar in the TA and control groups in males (**A**) and females (**B**). Legend as for Fig. 3.

The findings in Figs. 3 and 4 and their interpretation are described. + , positive value; -, negative value; TA, tooth agenesis; DB, disto-buccal cusp; DL, disto-lingual cusp; MB, mesio-buccal cusp; ML, mesio-lingual cusp.

Sex-specific differences included a general reduction in crown width in TA males, whereas in TA females, the reduction was localized to the mesial cervical width.

### Surface displacement in UM

In the TA group, the distolingual (DL) cusp was reduced in all dimensions in comparison to the control group, while the mesiolingual (ML) cusp and lingual groove showed enlargement in the lingual direction. These differences in the ML cusp were particularly pronounced in females, who exhibited a distally shifted and height-reduced ML cusp.

In both sexes, the mesiobuccal (MB) cusp and oblique groove were positioned higher, while the mesial pit and mesial marginal ridge were positioned lower in the TA group than in the control group. In addition, the triangular ridge of the distobuccal (DB) cusp was located more distally in the TA group. These differences in the DB cusp were more pronounced in females, extending lingually to the labial surface and mesial groove (*p *< 0.05; Table 2, Fig. 4).

### Dental morphospaces and calculation of TAShD and influence of allometry (size) on TAShD

**Fig. 5:**
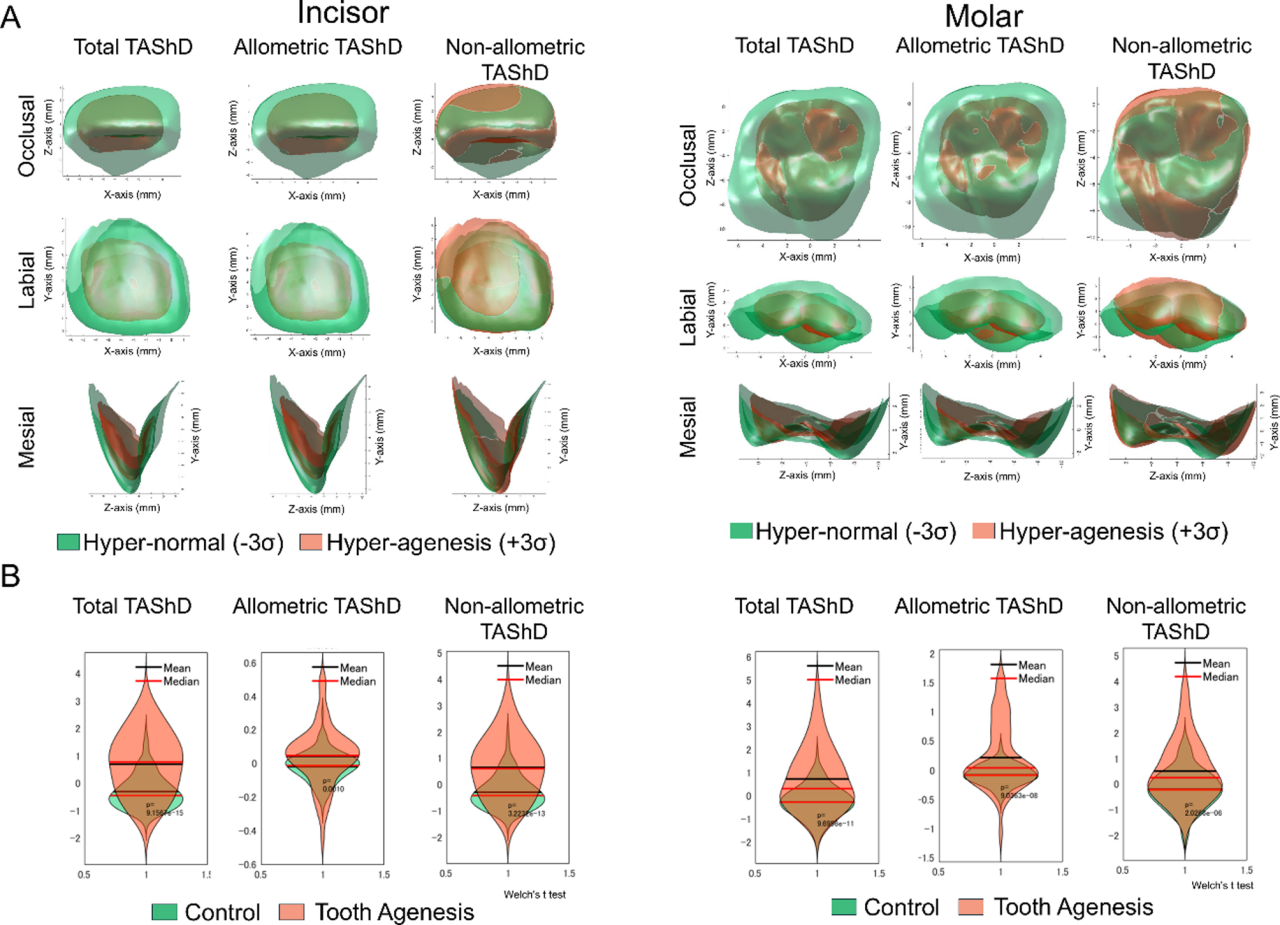
(**A**) Decomposition of TAShD into allometric TAShD and non-allometric TAShD; (**B**) Statistical differences of Total TAShD, allometric TAShD, and non-allometric TAShD between TA and control groups (Welch’s t-tests).

The first six PCs explained 80.4% of the sample variance in the maxillary right central incisor, and the first 10 significant PCs explained 80.8% of the variance in the maxillary right first molar. The TAShD analysis (Fig. 5) illustrated the 3D hyper-agenesis models of UI and UM, distinguishing between the allometric (size-related) and non-allometric components of shape variation. The TA group exhibited greater variation in both allometric and non-allometric TAShD than the control group, with significant differences observed between the control and TA groups (*P* < 0.05, Welch’s t-test).

**Fig. 6:**
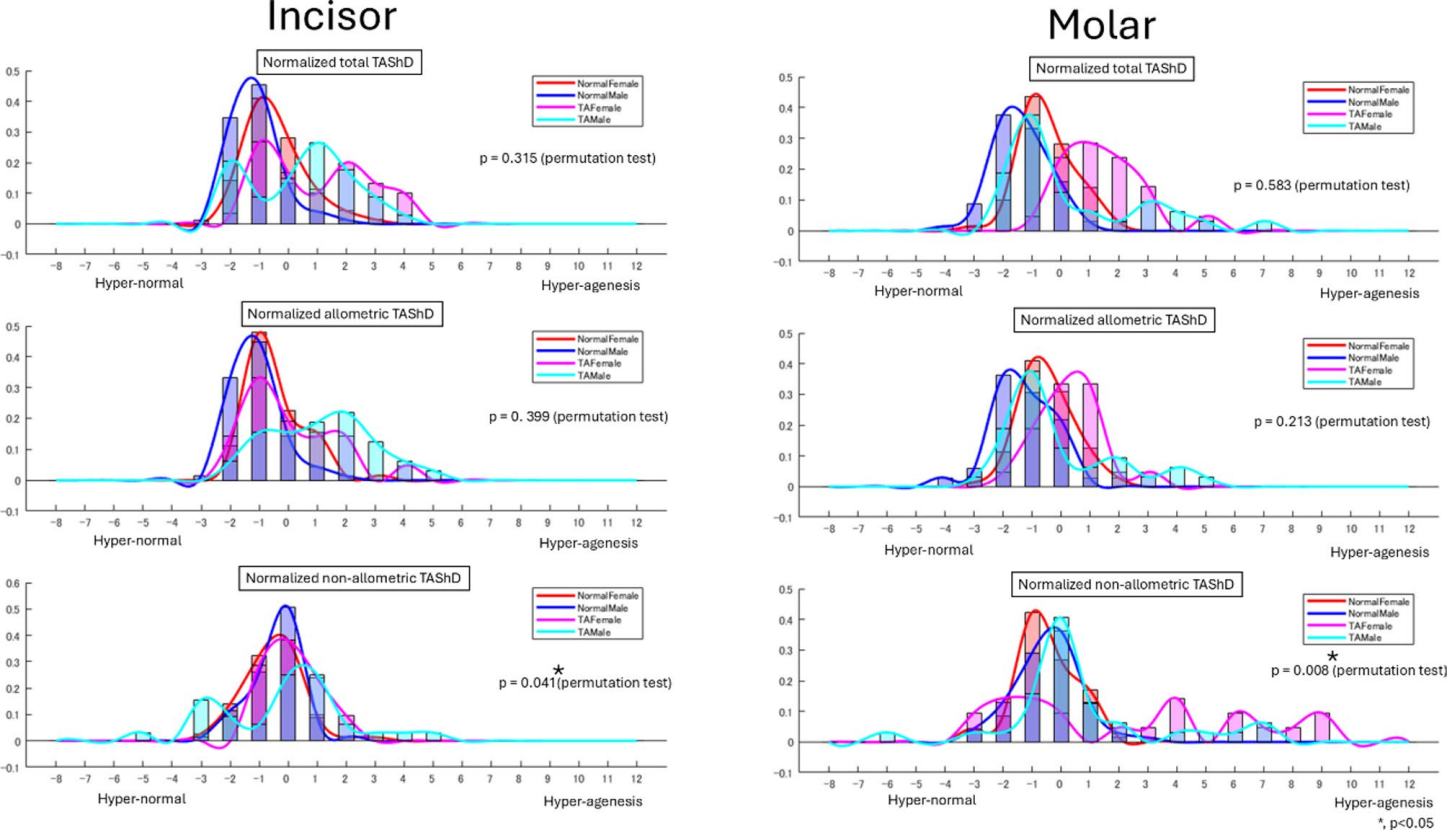
Distribution of TAShD, allometric TAShD, and non-allometric TAShD in Control female (red), Control male (blue), Tooth agenesis (TA) female (pink), and TA male (cyan). Distribution was normalized using the mean and standard deviation of the control female subgroup. The number of patients in each subgroup was normalized as 1 for comparison. The P-value for the permutation test is shown in the figure. * < 0.05.

The distribution of TAShD (Fig. 6) in the male and female TA subgroups exhibited similar bimodal patterns, suggesting the presence of distinct morphological tooth shape variations in TA. As one of these patterns resembled that of the control group, which exhibited a unimodal distribution, it is likely that multiple factors, including genetic influences, contribute to tooth agenesis.

The allometric TAShD revealed a bimodal distribution of UI for both sexes, whereas UM exhibited a unimodal distribution (Table 3). This suggests that size-related (allometric) characteristics associated with tooth agenesis were consistent across sexes and that the size of the incisors may be influenced by multiple factors. In contrast, the non-allometric TAShD demonstrated sex-dependent variation: females exhibited a unimodal distribution in UI but a bimodal distribution in UM, whereas males showed a bimodal distribution in UI and a unimodal distribution in UM. This indicates that shape-related factors differed between sexes, with molar shape in females and incisor shape in males being influenced by multiple factors.

**Table 3:** Summary of distribution of allometric tooth agenesis shape dimorphism (TAShD) and non-allometric TAShD in maxillary central incisor (UI) and maxillary first molar (UM).

	UI (Incisor)		UM (Molar)	
	Allometric (Size)	Non-Allometric (Shape)	Allometric (Size)	Non-Allometric (Shape)
Female	Bimodal	Unimodal	Unimodal	Multimodal
Male	Bimodal	Bimodal	Unimodal	Unimodal

The permutation test further confirmed that the TA–control differences significantly varied by sex, revealing significant sex differences in non-allometric TAShD. This indicates an interaction between the TA and sex. In contrast, no significant differences were observed in allometric TAShD, suggesting no interaction between sex- and size-related factors. These findings imply that size-independent (non-allometric) characteristics associated with tooth agenesis differ between the sexes, whereas size-related (allometric) characteristics remain consistent across the sexes (*p* < 0.05).

### Tooth agenesis patterns and related tooth shape

**Fig. 7:**
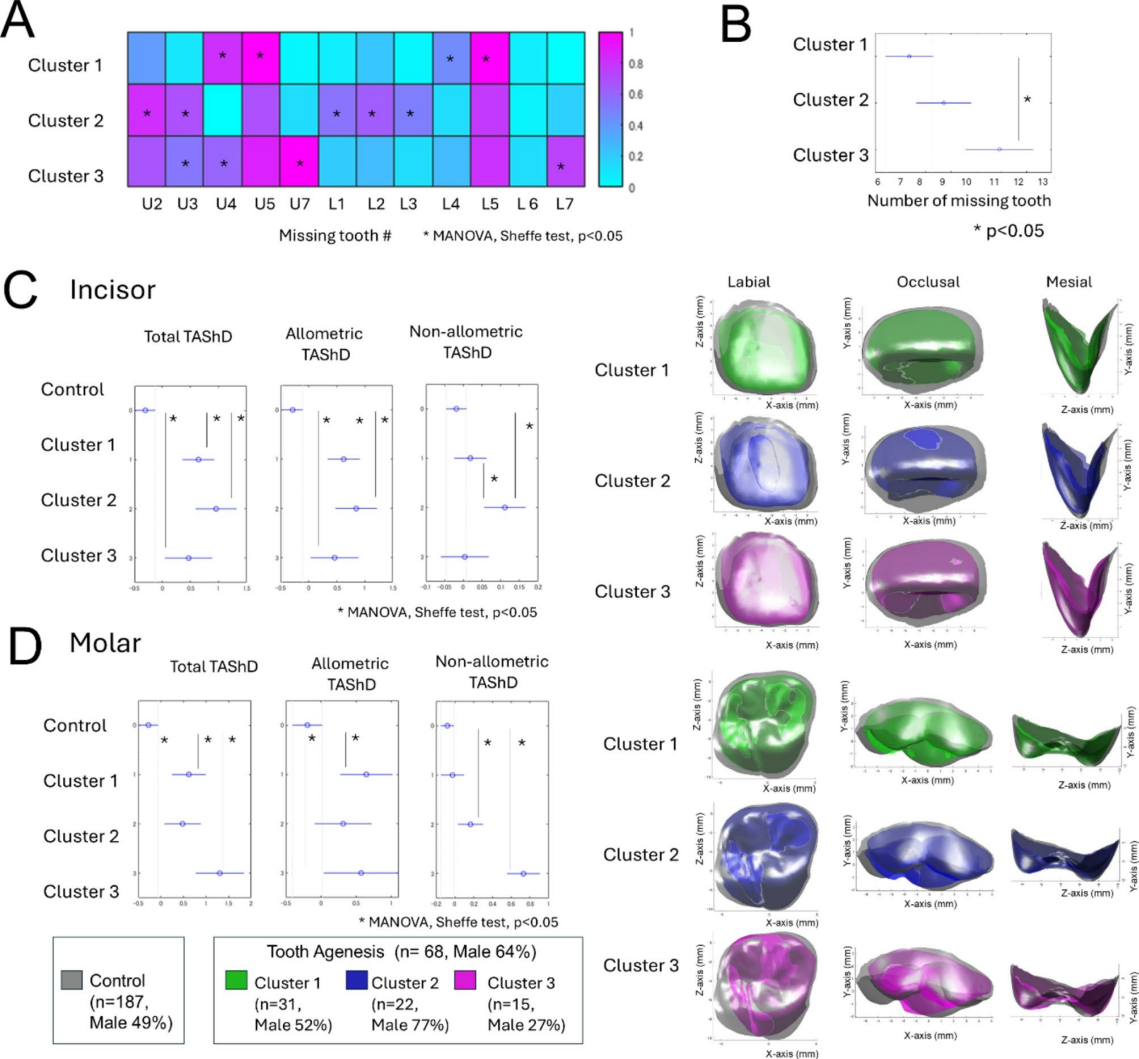
(**A**) Clustering results of the vector representation of missing teeth (Clusters 1, 2, and 3). In these vectors, each missing tooth in the maxilla and mandible is represented as '1 (pink),' while the presence of a tooth is represented as '0 (cyan).' Asterisk indicates a significantly greater number of missing teeth relative to at least one of the other two clusters (ANOVA, Scheffé’s test, p < 0.05). (**B**) The number of missing teeth in each cluster. Asterisk indicates significant differences among the three clusters (ANOVA) and between pairs of clusters (post-hoc Scheffé’s test, p < 0.05). (**C**) Differences in total TAShD, allometric TAShD, and non-allometric TAShD of the maxillary central incisors among the three clusters (ANOVA), and between pairs of clusters (post-hoc Scheffé’s test, p < 0.05). The right column displays the accentuated averaged shape of each cluster. The accentuation was performed using the following equation: T – C + T, where T represents the averaged shape of each cluster in the TA group, and C represents the averaged shape of the control group. (**D**) Similar to (**C**), but for the maxillary first molars.

The mathematical clustering method identified three distinct patterns of missing teeth in the samples (Fig. 7A). Table 4 provides a summary of the dominant missing teeth, sex distribution, and shape in each category. The details are as follows:

**Table 4:** Summary of each cluster.

	Typical pattern of MT	Average # of MT	Sex	UI		UM	
				Size	Shape	Size	Shape
Cluster 1	U4, U5, L4, L5	7	F = M	Small		Small	
Cluster 2	U2, U3	9	F < M	Small	Abnormal		
Cluster 3	U3, U4, U7, L7	11	F > M	Small		Small	Abnormal

MT: missing tooth, UI: upper central incisor, UM: upper first molar, F: female, M: male, U4, upper 1^st^ premolar, U5, upper 2^nd^ premolar; U2: upper lateral incisor, U3: upper canine, U7, upper 2^nd^ molar; L7, lower 2^nd^ molar.

### Dominant missing teeth

Cluster 1 was characterized by the absence of the maxillary and mandibular first and second premolars. Cluster 2 was characterized by the absence of maxillary and mandibular incisors and canines. Cluster 3 was characterized by the absence of maxillary canines, first premolars, and both maxillary and mandibular second molars. Cluster 3 showed a greater number of absences than cluster 2 (Fig. 7A).

### Sex distribution

Cluster 1 consisted of nearly equal numbers of males and females, Cluster 2 was predominantly composed of males (77%), and Cluster 3 was predominantly composed of females (73%) (Fig. 7B).

### Tooth shape and shape

Cluster 1 exhibited greater allometric TAShD for both UI and UM, whereas non-allometric TAShD showed no significant differences compared to the control group (Figs. 7C and D). This suggests that cluster 1 had a smaller tooth size, but the shape remained unaffected.

Cluster 2 exhibited greater values in both allometric and non-allometric TAShD for UI, whereas molars showed no significant differences in either allometric or non-allometric TAShD. This suggests that cluster 2 is primarily associated with differences in the size and shape of the maxillary central incisors.

Cluster 3 demonstrated significantly higher allometric TAShD values for the UI compared to the control group, while no significant differences were observed in non-allometric TAShD for UI. In contrast, both allometric and non-allometric TAShD values for molars were significantly higher than those in the control group. These findings suggest that cluster 3 represents a group with abnormal, smaller-shaped maxillary first molars and smaller maxillary central incisors.

## Discussion

The findings of this study offer valuable insights into the relationships between TA patterns, TA severity, and sex differences in 3D tooth morphology. Using homologous modeling techniques, we quantified the contributions of both size and shape to crown morphology and identified distinct TA patterns that influence dental form. Specifically, our results demonstrate that TA is associated with reduced tooth size and altered crown shape in UI and UM. The observed reduction in cingulum size in the UI and the diminished distolingual cusp in the UM among individuals with TA aligns with previous research, indicating that agenesis affects not only overall dental size but also specific morphological features^17–19^. These findings support the hypothesis that TA-related differences extend beyond mere tooth size reduction and involve shape modifications, potentially reflecting the underlying genetic and/or environmental mechanisms regulating morphogenesis. Moreover, the results provide important clues for addressing the two central research questions posed in this study, as outlined below:

*Question 1: Is it possible to integrate the TA variation of tooth size and morphology into a single continuous scale, and how do sex differences affect these relationships?*

A previous study^14^ proposed a multifactorial model with a continuous scale related to the tooth number and size, incorporating thresholds. This model was based on the observation that males more frequently exhibited supernumerary teeth and megadontia, whereas females more often had hypodontia and microdontia. Our data (Fig. 6 and Table 2) largely align with the previously proposed model, as tooth size variations in molars follow a normal distribution, and males and females exhibit similar TA variations (permutation test, *p* > 0.05). However, tooth shape exhibited greater variation than previously proposed, with shape-related factors differing between sexes. Specifically, molar shape in females and incisor shape in males showed a bimodal distribution, indicating the possible influence of multiple factors. This finding suggests that the multifactorial model proposed by Brook et al.^14^ may require some refinement, as the tooth shape does not follow a single continuous scale. Although the model is partially validated for tooth size, it may not fully account for variations in tooth shape. Our results indicate that the tooth shape is better represented within a multidimensional framework than in a single-dimensional model. The present study found that sex-related differences and TA independently influenced tooth shape; however, both factors had shared effects on size variation. This suggests that while TA and sexual dimorphism may arise from distinct developmental pathways, their combined impact on size may reflect a convergence in the regulatory mechanisms that govern overall tooth development. The absence of an interaction effect on shape differences indicated that the genetic or environmental pathways influencing TA and sexual dimorphism likely operate through separate morphogenetic processes.

Question* 2: How do different patterns of tooth agenesis relate to variations in tooth shape, and what insights can this provide into the mechanisms underlying tooth development?*

To examine the relationship between these shape variations and sex, the present study classified TA patterns and examined the relationships among TA patterns, sex variations, and 3D morphology in UI and UM. This idea is based on a previous study that showed distinct patterns of tooth agenesis^16,28,29^. Tooth formation is driven by epithelial-mesenchymal interactions with dental mesenchymal cells derived from multipotent cranial neural crest cells^30^. The molecular mechanisms underlying these processes involve complex signaling networks, including the Fgf, Bmp, Shh, and Wnt signaling pathways^3,31^. Disruptions in these tightly regulated cascades can result in defects in a specific tooth (i.e., the TA pattern), tooth size, and shape^32–34^. However, no study has examined the relationship between TA patterns and tooth shape.

In our study, we identified three distinct clusters of tooth agenesis patterns and their typical tooth shapes: Cluster 1 (premolar pattern), Cluster 2 (upper incisors and canines), and Cluster 3 (upper canines and upper and lower second molars). Given the hypothesis that TA patterns are not random but rather reflect the spatial and functional roles of specific genes during tooth development^35–37^, several candidate genes may underlie these patterns, which can be discussed based on previous meta-analyses including genetic studies and functional analyses with TA patterns^28,38^.

Cluster 1, characterized by premolar agenesis, is similar to reported TA patterns^28^, which is confirmed to be associated with MSX1 mutation, as this gene has been implicated in typical premolar agenesis^9,15,28^. Interestingly, Cluster 1 had a smaller tooth size, but its shape remained unaffected. MSX1 regulates the early stages of odontogenesis, particularly in the premolar and posterior tooth fields^39^. Recently, the molecular factors involved in presumptive incisor and molar regions have been further investigated, focusing on various stages of tooth development. This includes signaling molecules and homeobox genes expressed in both the epithelial and mesenchymal components of the developing tooth^40^. It is hypothesized that the early stages of odontogenesis may determine the presence of a tooth but not its shape. Future studies are expected to clarify the mechanisms underlying the reduction in size observed in these patterns. This cluster did not show sex differences, which is consistent with the reported autosomal dominant inheritance pattern of this gene.

In contrast, Cluster 2, involving the upper incisors and canines, is similar to the reported TA patterns^28^, which is confirmed to be related to mutations in EDA, EDAR, and EDARADD. These genes are involved in ectodermal dysplasia^41^ but also non-syndromic TA^42^. Previous data have shown that EDA mutations are specifically associated with a high prevalence of incisor agenesis, especially in maxillary central incisors, and this pattern extends to canines^43,44^. A missense mutation (p.Arg65Gly) in the EDA gene has been reported in a Chinese family with X-linked non-syndromic hypodontia^45^, another mutation (p.Gln358Glu) was detected in the affected members of an Indian family with X-linked hypodontia^43^. The high prevalence of Cluster 2 in males aligns with this finding. Cluster 2 was primarily associated with differences in the size and shape of the maxillary central incisors, with a smaller cingulum. In individuals with X-linked hypohidrotic ectodermal dysplasia, which is also related to EDA mutations, the incisors in the maxilla usually have a tapered and conical morphology^46^, but previous studies in Chinese populations^44,45^ stated that these tooth malformations were not observed in their study participants. Our results showed that the teeth in Cluster 2 do not exhibit a tapered or conical morphology, which is consistent with previous studies. Furthermore, interestingly, a common non-synonymous variant in EDAR, which is specific to Asian populations, has been shown to be associated with larger crown size, greater degree of shoveling, and double shoveling of upper incisors in Japanese and Korean populations^47,48^, indicating that EDAR is related to incisor morphology, which is not contradicted by our results.

Cluster 3, which includes agenesis of the upper canines and both the upper and lower second molars, was difficult to explain based on previous research^28^. A possible candidate gene is PAX9, as studies analyzing tooth agenesis patterns in individuals with PAX9 mutations have shown that the most common pattern involves missing lower second molars with a 100% prevalence^49,50^, which closely resembles the pattern observed in Cluster 3. This cluster also exhibited small incisors and morphologically altered molars, particularly those with reduced distolingual cusps (hypocones). These features are consistent with clinical observations of smaller tooth dimensions in PAX9-related cases, although agenesis of the anterior teeth has rarely been reported^51,52^. Furthermore, the present study found that Cluster 3 was more prevalent in females. While our search of the relevant literature did not identify any non-syndromic TA patterns specifically associated with females, several potential explanations merit consideration. One possibility is that PAX9 has been linked to Class II Division 2 malocclusion, which shows a higher prevalence in females, indicating a hormonal influence on genetic expression^53,54^.

Nonetheless, as the present study did not include a genetic analysis of the subjects, the scope of the discussion is limited. To better interpret our findings, it is necessary to include a genetic analysis and consider the potential influence of other genetic factors, such as AXIN2^55^ and WNT10A^56,57^, as well as environmental factors. The MSX1 rs8670 variant was associated with morphological variation, and recent evidence of compensatory interactions among maxillary incisors suggests that epigenetic and environmental factors modulate the phenotypic expression of this genetic variant^58^. Control of cusp morphology, including not only the shape but also the spatial arrangement of cusps, is a critical process in tooth development, enabling the formation of a wide range of tooth types. There is evidence that this process is regulated by enamel knots^59^. Intercuspal distances, which result from the folding of the internal enamel epithelium during odontogenesis following the formation of primary and secondary enamel knots, have been shown to be more influenced by epigenetic than genetic factors based on studies of monozygotic and dizygotic twins^60,61^. Given that our method using homologous modeling is well suited to describe detailed tooth shape, it could also be effectively applied to genetic or twin studies, which would provide valuable insights.

The application of homologous modeling and development of the TAShD metric provided a novel framework for quantifying dental morphological variations. By differentiating size-dependent and size-independent effects, this method allows for a more precise analysis of how TA and sex differences contribute to dental morphology. Future research could further refine this metric by incorporating additional anatomical landmarks and evaluating its applicability across diverse populations.

Despite these advancements, several limitations must be acknowledged. First, although the study employed a robust 3D modeling approach, the sample size and genetic background of the participants may affect the generalizability of the results. Especially, over the past few decades, a marked increase in the prevalence of tooth agenesis including third molars has been reported possibly due to changes in epidemiological patterns or improvements in diagnostic detection^29,62,63^. Future research with larger and more genetically diverse cohorts could help to validate these findings. Furthermore, while our analysis concentrated on the maxillary central incisor and first molar, future studies should explore other teeth in both the maxillary and mandibular arches to determine whether similar morphological trends are observed in both dental regions.

In conclusion, this study provides a comprehensive analysis of the interplay between TA, sexual dimorphism, and dental morphology, using advanced geometric morphometric techniques. The identification of distinct TA patterns and their corresponding morphological variations contributes to our understanding of tooth development and genetic influences on dental traits. Future investigations that integrate genetic data with 3D morphological analyses may further elucidate the mechanisms underlying these developmental processes.

## Conclusion

In the present study, homologous modeling of the UI and UM revealed that the tooth agenesis groups exhibited significantly smaller size and shape differences in comparison to the control group, with notable changes in the cingulum and rounder surfaces of the UI, as well as a smaller distolingual cusp in the UM. The study also demonstrated that sex and TA-associated factors independently influence shape differences but commonly affect size differences. Three missing tooth patterns were identified: Cluster 1 (absence of premolars), Cluster 2 (absence of incisors and canines), and Cluster 3 (absence of canines, first premolars, and second molars). Cluster 1 showed smaller tooth sizes without shape changes in UI and UM, Cluster 2 displayed both size and shape differences in the UI, and Cluster 3 exhibited smaller and abnormal shapes, primarily in the UM. These findings emphasize the distinct missing tooth patterns and their associated morphological variations.

## Electronic supplementary material

Below is the link to the electronic supplementary material.

Supplementary Material 1

## Data Availability

The data that support the findings of this study are available from the corresponding author, CT, upon reasonable request.
