# Supplementary material for: Three-dimensional tooth morphology in patients with tooth agenesis and its association to agenesis pattern, severity, and sex
Source: Sci Rep. 2025 Aug 1;15:28119. doi: 10.1038/s41598-025-11034-6 (PMC12317028; doi:10.1038/s41598-025-11034-6)
Supplement: Supplementary file 1 — Supplementary Material 1 [file 41598_2025_11034_MOESM1_ESM.docx]

Supporting information

**Three-dimensional tooth morphology in patients with tooth agenesis and its association to agenesis pattern, severity, and sexSupplementary Text S1. Preliminary experiments for establishing a new coordinate axis setting method and examination of its accuracy**

To standardize the coordinate axes for each tooth, we developed a novel method for defining the tooth coordinate axes using 3D surface data of the crowns of the maxillary right central incisors and first molars.

This method was validated using pre-treatment dental models and cone-beam computed tomography (CBCT) images from 12 randomly selected patients (6 males, 6 females; mean age, 11.8 ± 1.8 years; age range, 6–18 years) who visited the Department of Orthodontics at Osaka University Dental Hospital between 2012 and 2019.

The inclusion criteria were as follows: existence of CBCT data and dental models; no medical or dental history (including treatment or trauma); no reports of bruxism or parafunctional habits; no microdontia or tooth agenesis; and no restorations, caries, tooth wear, gingivitis, or gingival recession on the maxillary right central incisor or first molar. Exclusion criteria included patients whose teeth could not be fully scanned due to crowding and dental models with surface roughness or air bubbles. Intraoral photographs and panoramic radiographs were used to assess the presence of restorations, caries, gingivitis, and gingival recession. The Smith and Knight Tooth Wear Index (TWI) {Smith, 1984 #2159} was used, and only patients with TWI scores of 0 (no loss of enamel surface characteristics and no change in contour) or 1 (loss of enamel characteristics, but minimal loss of contour) were included.

Maxillary impressions were obtained using alginate (Aroma Fine Plus Fast Set, GC Corporation, Tokyo, Japan) and dental models were fabricated using dental hard stone (New Plastone II White, GC Corporation, Japan). These models were scanned at an accuracy of 10 μm using a 3D optical scanner (Maestro 3D Dental Scanner (MDS 350), AGE Solutions, Italy). The resulting 3D surface data were transferred to a measurement software program (Maestro 3D Dental Studio, AGE Solutions, Italy), extracted as STL files, and imported into a homologous modeling software program (HBM-Rugle, Medic Engineering Co., Kyoto, Japan). On a 23-inch monitor (DELL U2312HM, resolution: 1920 × 1080), the crown surfaces of the maxillary right central incisor and the first molar were visually delineated at the gingival margin using digital models. The 3D surface data were saved in STL files.

CBCT scans were acquired at Osaka University Dental Hospital to evaluate the position of the incisors and potential root resorption in the adjacent teeth. Imaging parameters included 80 kVp tube voltage, 2.0 mA tube current, and a voxel size of 0.39 × 0.39 × 0.39 mm (Alphard-3030; Asahi Roentgen Ind. Co., Ltd., Kyoto). Patients with maxillary right central incisors and first molars with incomplete root formation or root resorption were excluded.

The crown surface data for the maxillary right central incisors and first molars were extracted following the same procedure. Using a 3D medical image processing software program (Materialize Mimics, Materialise HQ Technologielaan, Belgium), the entire tooth, including the crown and root, was automatically segmented by applying a threshold that distinguished the tooth from the surrounding tissues in the CBCT images. Manual corrections were made to remove outliers and add any missing regions. The resulting 3D surface data for each tooth were saved as STL files.

Anatomical landmarks were manually identified on the 3D tooth models derived from CBCT using the homologous modeling support software program, and tooth axes were defined accordingly (Supplementary Fig. S1).

For the maxillary right central incisor, the tooth axis was defined as a straight line connecting the center of the incisal edge and the root apex (Supplementary Fig. S2). The origin was set to the mesial point of the incisal edge. The X-axis was defined as the line from the origin to the distal point of the incisal edge, representing the mesiodistal direction. The Y-axis is defined as a line through the origin parallel to the tooth axis. The Z-axis was determined using a right-handed coordinate system based on the X- and Y-axes.

For the maxillary right first molar, the tooth axis was defined as the line connecting the centroid of the apices of the three roots to the central fossa (Supplementary Fig. S2). The origin was defined as the junction of the buccal groove and occlusal surface. The Z-axis, corresponding to the buccolingual direction, was defined as the line passing through the origin and the central pit. The Y-axis was aligned with the tooth axis through the origin and the X-axis was defined using a right-handed coordinate system based on the Y- and Z-axes.

Using the homologous modeling software program, the coordinate-defined 3D tooth models (W_i) were superimposed onto the crown surface data from the same patient's dental model (C_i) via the least-squares method (Supplementary Fig. S3).

For homologous modeling, we utilized predefined template models for the maxillary right central incisor and first molar, consisting of 1,889 and 1,842 data points, respectively. Anatomical landmarks were assigned to these templates using the method described in the Supplementary Text S2. Template models were fitted to each patient's 3D crown data by aligning the landmarks, and the results were averaged using the HBM software program to create average crown shape models (M′_Ave) (Supplementary Fig. S2). The coordinate axes were then assigned to all patient crown models (C_j) by superimposing M′_Ave onto each C_j using the least-squares method (Supplementary Fig. S2).

To evaluate the accuracy of the proposed coordinate axis setting method, we calculated the rotation angle of the tooth axis by superimposing the crown model with the newly assigned coordinate system onto the reference crown model (C′_i), using the least-squares method. Additionally, crown length (C) and root length (R) along the Y-axis were measured in W_i with the true coordinate system. The tangent of the rotation angle (φ) was calculated from the tooth axis direction components along the X- and Z-axes (Supplementary Fig. S3).

The results, including rotation angles and errors at the cervical and root apex regions of the maxillary right central incisor and first molar, are presented in Supplementary Table S1. The mean tooth axis rotation error ranged from 2.2° to 2.6°, with average cervical region errors below 0.3 mm. These errors were smaller than the CBCT voxel size, indicating that the proposed method was accurate and reliable for determining the tooth axis.

**Supplementary Text S2. Verification of the accuracy of the identification of anatomical landmarks**

To define reliable anatomical landmarks, intra- and inter-class correlation coefficients (ICCs) and minimal detectable change with 95% confidence (MDC95) were calculated from the coordinate values of the landmarks, as described below.

Pre-treatment dental models from 10 patients (4 males and 6 females; mean age: 14.9 ± 8.5 years) were randomly selected from individuals who visited the Department of Orthodontics at Osaka University Dental Hospital between 2012 and 2019. The selection and exclusion criteria were consistent with those outlined in Supplementary Text S1.

A total of 21 anatomical landmarks on the maxillary right central incisor and 34 on the maxillary right first molar were identified with reference to previous studies (Supplementary Figs. S4 and S5 and Tables S2-1 and S2-2). The landmarks were visually identified by two orthodontists (Examiners A and B), each with over seven years of clinical experience, through inspection of the 3D surface images. Landmark identification was performed twice by each examiner on two separate occasions with a three-day interval. The landmarks were digitized using a computer mouse and a homologous modeling software program, and the coordinate values were exported as comma-separated value (CSV) files.

The intraclass correlation coefficient (ICC(1,2)) was calculated from the coordinate values of each examiner's first and second identifications, while the interclass correlation coefficient (ICC(2,2)) was calculated from the coordinate values between Examiners A and B. ICC values represent relative reliability and were interpreted as follows: ICC < 0.50 = "poor," ICC 0.50 to < 0.75 = "moderate," ICC 0.75 to < 0.90 = "good," and ICC ≥ 0.90 = "excellent" reliability, based on the criteria of Koo et al.^2^.

Landmarks with both intra- and inter-examiner ICCs below 0.75 were excluded. As a result, 16 anatomical landmarks on the maxillary right central incisor and 17 on the maxillary right first molar were deemed reliable and were included in the analysis (Supplementary Tables S3 and S4). The absolute mean differences in coordinate values at landmark points ranged from 0.14 to 0.24 mm, with ICC values ranging from 0.91 to 0.98. The average MDC95 values were 0.34–0.39 mm for the X-axis, 0.23–0.40 mm for the Y-axis, and 0.24–0.38 mm for the Z-axis (Supplementary Table S5).

**Supplementary Text S3. Statistical analysis**

The analysis was conducted in four phases: 1) the first focused on surface displacement in Cartesian coordinates (x, y, z), and 2) the second utilized a newly defined coordinate system, termed dental morphospace. Within the morphospace, 3) the calculation of TAShD was performed. Then, 4) the patterns of the missing teeth were determined, and the tooth shape in each pattern was examined. The details are as follows:

*1) Surface displacement*

To standardize the orientation of UI and UM, a Procrustes analysis was performed, which involved translating, rotating, and scaling homologous models. The scaling was based on the centroid size (CS), calculated as the square root of the sum of the squared distances between each point and the center of gravity of the homologous models for both UI and UM. Subsequently, the arithmetic means of the coordinate values for each corresponding point on the wire mesh were computed to generate averaged 3D tooth images for the TA female, TA male, control female, and control male subgroups. Surface displacement was quantitatively assessed along the X-, Y-, and Z-axes using two methods. First, displacement (Control to TA for each sex) was calculated for 1,889 points for UI and 1,842 points for UM on each mesh for the TA and control groups, with the displacement expressed in millimeters and visualized using color coding as a distance map. Second, the arithmetic means of the coordinate values for each corresponding point were analyzed for significant differences between subgroups using a two-sample t-test ^26^. A significance probability map for the X-, Y-, and Z-values was generated to represent these significances visually.

*2) Dental morphospace*

To determine the dental morphospace for each UI and UM, we reduced dimensionality by performing a principal component analysis (PCA) for the coordinates of the aforementioned homologous models in UI and UM. Significant principal components (PCs) were determined using a scree plot analysis. PCs whose cumulative contribution ratio was > 80% were used as dental morphospace in the subsequent calculations.

*3) Calculation of TAShD and influence of allometry(size) on TAShD*

To examine allometry, which describes how the morphometric characteristics of teeth change with tooth size ^27^, the TAShD of the individual tooth was measured by projection of the individual dental configurations onto an axis connecting the vector between the average configurations of control and agenesis groups in the tooth morphospace using the following equation:

$TAShD(\vec{F_{i}}) = \frac{( \vec{F_{i}}\cdot\vec{F_{(a-c)}})}{{|\vec{F_{(a-c)}}|}^{2}}$

where $\vec{F_{i}}$is the vector in the tooth morphospace corresponding to an individual tooth *i*, and $\vec{F_{(a-c)}}$ is the vector between the agenesis and control configurations (agenesis minus control). If TAShD < -1, the tooth is considered to show hyperagenesis, and if TAShD > 1, the tooth is considered to be hypernormal.

Furthermore, TAShD measures were mathematically decomposed into allometric and non-allometric components. Variations in TAShD due to an individual’s size (allometric) and variations that were independent of size (non-allometric) were examined in the overall variation in TAShD in each group with/without TA using a multivariate regression analysis. CS was used as a measure of an individual’s size. The allometric variation in TAShD was calculated by regressing the shapes in the tooth morphospace on CS and projecting the estimated values from this regression onto the vector of differences between the TA and control groups. The non-allometric component of TAShD was acquired by regressing the shapes in the tooth morphospace on CS and then projecting the residualized tooth coordinates on the TA-control difference vector calculated with these residuals ^13^.

Welch’s test was conducted to examine whether the TAShD (total, allometric, and non-allometric components) differed between the TA and control groups. To test whether these observed differences in TAShD (total, allometric, and non-allometric components) between the TA and control groups differed significantly between sexes, permutation tests were conducted as a randomization test. In each iteration of randomization, sex labels were randomly reassigned to individuals while preserving the TA/control group assignment and number in the TA and control groups for each sex. A total of 1000 randomized samples were generated for each permutation test.

*4) Tooth agenesis patterns.*

The missing teeth were identified using panoramic radiography. Each missing tooth in the maxilla and mandible was coded as “1,” whereas the presence of a tooth was coded as “0,” generating a vector representation of missing teeth. To reduce the dimensionality of the vector, the missing tooth data from the left and right sides were combined by mirroring the left-side missing teeth onto the right. If a tooth was missing on either side, it was assigned a value of “1” in the vector. Missing tooth patterns were classified using a k-means clustering analysis. The number of clusters was determined using the elbow method. An analysis of variance (ANOVA) was used to examine differences among clusters in allometric and non-allometric TAShD as well as the number of missing teeth. The average shape of the teeth in each group was determined.

**Supplementary Table S1. Averaged rotation angles, errors in the cervical region, and errors in the root apex of the maxillary right central incisor and first molar**

|  | Central incisor | |  | First molar | |
| --- | --- | --- | --- | --- | --- |
|  | X | Z |  | X | Z |
| Rotation angle (°) | 2.2 | 2.4 |  | 2.6 | 2.6 |
| Error at cervical (mm) | 0.3 | 0.3 |  | 0.2 | 0.2 |
| Error at apex (mm) | 0.8 | 0.9 |  | 0.8 | 0.9 |

**Supplementary Table S2-1. Definitions of landmarks on the maxillary right central incisor**

| Landmark | Definition |
| --- | --- |
| 1 (origin) | Mesioincisal angle point |
| 2 | Mesiolabial developmental groove in labioincisal edge |
| 3 | Distolabial developmental groove in labioincisal edge |
| 4 | Top of distoincisal angle |
| 5 | Distoincisal angle |
| 6 | Linguoincisal edge corresponding to 3 |
| 7 | Linguoincisal edge corresponding to 2 |
| 8 | Mesial contact point |
| 9 | End point of mesiolabial developmental groove |
| 10 | End point of distolabial developmental groove |
| 11 (excluded) | Mesial gingival margin of the labial surface |
| 12 | Central gingival margin of the labial surface |
| 13 (excluded) | Distal gingival margin of the labial surface |
| 14 | Coronally point of distal marginal ridge |
| 15 | Cervix point of distal marginal ridge |
| 16 | Cingulum |
| 17 | Cervix point of mesial marginal ridge |
| 18 | Coronally point of mesial marginal ridge |
| 19 | Distal gingival margin of the lingual surface |
| 20 (excluded) | Central gingival margin of the lingual surface |
| 21 | Mesial gingival margin of the lingual surface |

**Supplementary Table S2-2. Definitions of landmarks on the maxillary right first molar.**

| Landmark | Definition |
| --- | --- |
| 1 (excluded) | Most mesial junction of the occlusal groove with the occlusal surface. |
| 2 (excluded) | Mesial end of mesiobuccal triangular developmental groove |
| 3 (excluded) | Mesiobuccal cusp tip |
| 4 (origin) | Junction of the buccal groove with the occlusal surface |
| 5 | Distobuccal cusp tip |
| 6 (excluded) | Distal end of distobuccal triangular developmental groove |
| 7 (excluded) | Distal end of distolingual triangular groove |
| 8 (excluded) | Distolingual cusp tip |
| 9 (excluded) | Halfway between 8 and 10 |
| 10 | Lingual end of distal oblique groove |
| 11 | Mesiolingual cusp tip |
| 12 | Mesial end of mesial groove |
| 13 | Distal triangular fossa |
| 14 (excluded) | Central fossa |
| 15 (excluded) | Mesial triangular fossa |
| 16 (excluded) | Halfway between 2 and 24 |
| 17 (excluded) | Buccal pit (end of buccal groove) |
| 18 (excluded) | Curvature of distobuccal cusp |
| 19 | Curvature of distolingual cusp |
| 20 | End of lingual groove |
| 21 (excluded) | Curvature of mesiolingual cusp |
| 22 | Curvature of mesiolingual cusp (mesial surface) |
| 23 (excluded) | Mesial contact point |
| 24 (excluded) | Mesial gingival margin corresponding to 2 |
| 25 | Buccal gingival margin corresponding to 3 |
| 26 | Buccal gingival margin corresponding to 17 |
| 27 (excluded) | Buccal gingival margin corresponding to 5 |
| 29 | Distal contact point |
| 30 | Distal lingual gingival margin corresponding to 8 |
| 31 | Lingual gingival margin corresponding to 8 |
| 32 | Lingual gingival margin corresponding to 20 |
| 33 | Lingual gingival margin corresponding to 11 |
| 34 (excluded) | Mesial gingival margin corresponding to 11 |

**Supplementary Table S3. The results of** **intra- and inter- class correlation coefficients (ICCs) in the anatomical landmarks on maxillary right central incisors**

| Landmark No. | ICC(1,2) | | | | | | | | | | | | |  | ICC(2,2) | | | | | | | Included  landmarks | |  |
| --- | --- | --- | --- | --- | --- | --- | --- | --- | --- | --- | --- | --- | --- | --- | --- | --- | --- | --- | --- | --- | --- | --- | --- | --- |
|  | Examiner A | | | | | |  | Examiner B | | | | | |  |  | | |  | |  | |  | |  |
|  | x | | y | | z | |  | x | | y | | z | |  | x | | | y | | z | |  | |  |
| 1 | 1.00 | | 1.00 | | 0.99 | |  | 1.00 | | 1.00 | | 1.00 | |  | 1.00 | | | 1.00 | | 1.00 | | ✓ | |  |
| 2 | 1.00 | | 1.00 | | 1.00 | |  | 0.99 | | 1.00 | | 0.99 | |  | 1.00 | | | 1.00 | | 1.00 | | ✓ | |  |
| 3 | 1.00 | | 1.00 | | 0.99 | |  | 1.00 | | 1.00 | | 0.99 | |  | 1.00 | | | 1.00 | | 1.00 | | ✓ | |  |
| 4 | 1.00 | | 1.00 | | 0.99 | |  | 1.00 | | 1.00 | | 0.97 | |  | 1.00 | | | 1.00 | | 0.99 | | ✓ | |  |
| 5 | 0.88 | | 0.98 | | 1.00 | |  | 1.00 | | 1.00 | | 1.00 | |  | 0.98 | | | 0.99 | | 1.00 | | ✓ | |  |
| 6 | 0.97 | | 0.99 | | 1.00 | |  | 1.00 | | 1.00 | | 1.00 | |  | 0.99 | | | 1.00 | | 1.00 | | ✓ | |  |
| 7 | 0.96 | | 0.99 | | 1.00 | |  | 0.99 | | 1.00 | | 1.00 | |  | 0.99 | | | 0.99 | | 1.00 | | ✓ | |  |
| 8 | 0.85 | | 0.99 | | 1.00 | |  | 1.00 | | 1.00 | | 1.00 | |  | 0.96 | | | 1.00 | | 0.99 | | ✓ | |  |
| 9 | 0.96 | | 0.95 | | 0.94 | |  | 0.98 | | 0.96 | | 0.96 | |  | 0.98 | | | 0.96 | | 0.94 | | ✓ | |  |
| 10 | 0.99 | | 0.96 | | 0.97 | |  | 0.99 | | 0.97 | | 0.95 | |  | 0.99 | | | 0.97 | | 0.96 | | ✓ | |  |
| 11 | 0.88 | | 0.99 | | 0.44 | |  | 0.98 | | 0.99 | | 0.90 | |  | 0.72 | | | 0.95 | | 0.23 | |  | |  |
| 12 | 0.87 | | 0.97 | | 0.97 | |  | 0.97 | | 0.96 | | 0.96 | |  | 0.95 | | | 0.99 | | 0.99 | | ✓ | |  |
| 13 | | 0.98 | | 0.98 | | 0.62 | |  | 1.00 | | 0.99 | | 0.87 | | |  | 0.98 | | 0.97 | | 0.34 | |  | |
| 14 | | 1.00 | | 0.98 | | 1.00 | |  | 1.00 | | 0.98 | | 1.00 | | |  | 0.99 | | 0.99 | | 0.99 | | ✓ | |
| 15 | | 0.99 | | 0.95 | | 0.98 | |  | 1.00 | | 0.93 | | 0.92 | | |  | 0.98 | | 0.87 | | 0.62 | |  | |
| 16 | | 0.99 | | 0.95 | | 0.97 | |  | 0.99 | | 0.98 | | 0.97 | | |  | 1.00 | | 0.97 | | 0.93 | | ✓ | |
| 17 | | 0.90 | | 0.95 | | 0.97 | |  | 0.99 | | 0.96 | | 0.91 | | |  | 0.91 | | 0.88 | | 0.81 | | ✓ | |
| 18 | | 0.98 | | 0.96 | | 1.00 | |  | 0.99 | | 0.96 | | 0.99 | | |  | 0.99 | | 0.99 | | 1.00 | | ✓ | |
| 19 | | 0.99 | | 0.97 | | 0.93 | |  | 0.99 | | 0.98 | | 0.96 | | |  | 0.99 | | 0.96 | | 0.90 | | ✓ | |
| 20 | | 1.00 | | 0.58 | | 1.00 | |  | 1.00 | | 0.99 | | 1.00 | | |  | 0.99 | | 0.91 | | 0.99 | |  | |
| 21 | | 0.55 | | 0.96 | | 0.87 | |  | 0.85 | | 0.98 | | 0.98 | | |  | 0.92 | | 0.75 | | 0.87 | |  | |
|  |  | |  | |  | |  |  | |  | |  | |  |  | | |  | |  | |  | |  |

✓: Anatomical landmarks adopted as reliable landmarks with ICC≧0.75

**Supplementary Table S4. The results of intra- and inter- class correlation coefficients (ICCs) in the anatomical landmarks on maxillary right first molars.**

| Landmark No. | ICC(1,2) |  |  |  |  |  | ICC(2,2) |  |  | Included landmarks |
| --- | --- | --- | --- | --- | --- | --- | --- | --- | --- | --- |
|  | Examiner A | | | Examiner B | | |  |  |  |  |
|  | x | y | z | x | y | z | x | y | z |  |
| 1 | 0.73 | 0.23 | 0.95 | 0.9 | 0.65 | 0.71 | 0.54 | 0.27 | 0.62 |  |
| 2 | 0.72 | 0.66 | 0.59 | 0.88 | 0.96 | 0.95 | 0.54 | 0.86 | 0.83 |  |
| 3 | 0.8 | 0.95 | 0.96 | 0.13 | 0.82 | 0.94 | 0.77 | 0.96 | 0.96 |  |
| 4 | 0.99 | 1.00 | 0.86 | 0.98 | 0.96 | 0.76 | 0.92 | 0.97 | 0.88 | ✓ |
| 5 | 0.98 | 0.97 | 0.80 | 0.95 | 1.00 | 0.83 | 0.99 | 0.97 | 0.62 | ✓ |
| 6 | 0.97 | 0.87 | -0.11 | 0.98 | 0.67 | 0.70 | 0.96 | 0.61 | 0.41 |  |
| 7 | 0.95 | 0.76 | 0.42 | 0.97 | 0.86 | 0.70 | 0.95 | 0.91 | 0.79 |  |
| 8 | 0.94 | 0.59 | 0.79 | 0.99 | 0.94 | 0.9 | 0.98 | 0.94 | 0.67 |  |
| 9 | 0.97 | 0.99 | 0.98 | 0.9 | 0.89 | 0.91 | 0.92 | 0.98 | 0.94 |  |
| 10 | 0.97 | 0.98 | 0.96 | 0.93 | 0.95 | 0.98 | 0.99 | 0.99 | 0.96 | ✓ |
| 11 | 0.91 | 0.99 | 0.84 | 0.76 | 0.94 | 0.81 | 0.76 | 0.96 | 0.81 | ✓ |
| 12 | 0.76 | 0.94 | 0.91 | 0.75 | 0.82 | 0.93 | 0.76 | 0.75 | 0.75 | ✓ |
| 13 | 0.92 | 0.77 | 0.96 | 0.94 | 0.91 | 0.92 | 0.92 | 0.92 | 0.97 | ✓ |
| 14 | 0.85 | 0.88 | 0.39 | 0.8 | 0.93 | 0.82 | 0.79 | 0.89 | 0.61 |  |
| 15 | 0.91 | 0.9 | 0.91 | 0.68 | 0.9 | 0.21 | 0.69 | 0.95 | 0.54 |  |
| 16 | 0.93 | 0.96 | 0.91 | 0.27 | 0.84 | 0.43 | 0.87 | 0.92 | 0.70 |  |
| 17 | 0.93 | 0.61 | 0.97 | 0.98 | 0.13 | 0.97 | 0.98 | 0.43 | 0.94 |  |
| 18 | 0.97 | 0.89 | 0.77 | 0.99 | 0.94 | 0.66 | 0.97 | 0.9 | 0.67 |  |
| 19 | 0.99 | 0.95 | 0.96 | 0.99 | 0.98 | 0.98 | 0.99 | 0.97 | 0.93 | ✓ |
| 20 | 0.91 | 0.81 | 0.96 | 0.96 | 0.92 | 0.98 | 0.95 | 0.83 | 0.98 | ✓ |
| 21 | 0.97 | 0.87 | 0.97 | 0.9 | 0.49 | 0.95 | 0.94 | 0.37 | 0.95 |  |
| 22 | 0.83 | 0.75 | 0.75 | 0.92 | 0.85 | 0.90 | 0.9 | 0.81 | 0.85 | ✓ |
| 23 | 0.98 | 0.98 | 0.93 | 0.94 | 1.00 | 0.89 | 0.93 | 0.99 | 0.67 |  |
| 24 | 0.67 | 0.93 | 0.88 | 0.84 | 0.99 | 0.94 | 0.89 | 0.99 | 0.95 |  |
| 25 | 0.79 | 0.97 | 0.94 | 0.9 | 0.99 | 0.98 | 0.97 | 1.00 | 0.99 | ✓ |
| 26 | 0.93 | 0.98 | 0.98 | 0.94 | 1.00 | 0.99 | 0.92 | 1.00 | 0.99 | ✓ |
| 27 | 0.82 | 0.97 | 0.67 | 0.76 | 0.97 | 0.71 | 0.96 | 0.99 | 0.96 |  |
| 28 | 0.95 | 0.92 | 0.76 | 0.94 | 0.92 | 0.77 | 0.97 | 0.98 | 0.86 | ✓ |
| 29 | 0.99 | 0.96 | 0.86 | 0.98 | 0.98 | 0.95 | 0.99 | 0.99 | 0.9 | ✓ |
| 30 | 0.97 | 0.99 | 0.91 | 0.97 | 0.97 | 0.91 | 0.99 | 0.99 | 0.97 | ✓ |
| 31 | 0.92 | 0.98 | 0.98 | 0.88 | 0.97 | 0.96 | 0.98 | 0.99 | 0.99 | ✓ |
| 32 | 0.89 | 0.97 | 0.86 | 0.92 | 0.98 | 0.96 | 0.93 | 0.99 | 0.97 | ✓ |
| 33 | 0.9 | 0.98 | 0.98 | 0.91 | 0.99 | 0.99 | 0.8 | 0.95 | 0.97 | ✓ |
| 34 | 0.77 | 0.73 | 0.69 | 0.93 | 0.94 | 0.92 | 0.8 | 0.83 | 0.69 |  |

* Denotes the anatomical landmarks adopted as reliable landmarks with ICC ≥ 0.75.

**Supplementary Table S5. Mean, maximum, and minimum values and standard deviations for measurements in the X-, Y- and Z- directions for maxillary central incisors (UI) and first molars (UM).**

|  |  |  |  |  | Examiner A | | | |  | Examiner B | | | |  | Inter-examiners | | | |
| --- | --- | --- | --- | --- | --- | --- | --- | --- | --- | --- | --- | --- | --- | --- | --- | --- | --- | --- |
|  |  |  |  |  | Mean | Max | Min | SD |  | Mean | Max | Min | SD |  | Mean | Max | Min | SD |
| UI | Abs. diff | |  | x | 0.24 | 0.43 | 0.07 | 0.10 |  | 0.20 | 0.30 | 0.07 | 0.07 |  | 0.22 | 0.34 | 0.07 | 0.07 |
|  | (mm) | |  | y | 0.25 | 0.42 | 0.08 | 0.10 |  | 0.22 | 0.44 | 0.05 | 0.12 |  | 0.24 | 0.40 | 0.07 | 0.11 |
|  |  |  |  | z | 0.14 | 0.29 | 0.06 | 0.06 |  | 0.14 | 0.27 | 0.06 | 0.06 |  | 0.14 | 0.22 | 0.07 | 0.05 |
|  | ICCs |  |  | x | 0.96 | 1.00 | 0.85 | 0.05 |  | 0.99 | 1.00 | 0.97 | 0.01 |  | 0.98 | 1.00 | 0.91 | 0.02 |
|  |  |  |  | y | 0.98 | 1.00 | 0.95 | 0.02 |  | 0.98 | 1.00 | 0.96 | 0.02 |  | 0.98 | 1.00 | 0.88 | 0.03 |
|  |  |  |  | z | 0.98 | 1.00 | 0.93 | 0.02 |  | 0.98 | 1.00 | 0.91 | 0.02 |  | 0.97 | 1.00 | 0.81 | 0.05 |
|  | MDC95 | |  | x | 0.39 | 0.76 | 0.18 | 0.17 |  | 0.34 | 0.55 | 0.16 | 0.10 |  | 0.37 | 0.76 | 0.16 | 0.15 |
|  | (mm) | |  | y | 0.40 | 0.55 | 0.16 | 0.13 |  | 0.39 | 0.70 | 0.12 | 0.18 |  | 0.39 | 0.70 | 0.12 | 0.16 |
|  |  |  |  | z | 0.24 | 0.42 | 0.11 | 0.10 |  | 0.24 | 0.45 | 0.08 | 0.11 |  | 0.24 | 0.45 | 0.08 | 0.11 |
| UM | Abs. diff | |  | x | 0.25 | 0.56 | 0.03 | 0.13 |  | 0.22 | 0.41 | 0.04 | 0.11 |  | 0.23 | 0.40 | 0.07 | 0.09 |
|  | (mm) |  |  | y | 0.14 | 0.37 | 0.01 | 0.10 |  | 0.14 | 0.30 | 0.02 | 0.08 |  | 0.14 | 0.34 | 0.02 | 0.08 |
|  |  |  |  | z | 0.21 | 0.49 | 0.04 | 0.13 |  | 0.22 | 0.41 | 0.01 | 0.11 |  | 0.22 | 0.38 | 0.02 | 0.10 |
|  | ICCs |  |  | x | 0.92 | 0.99 | 0.76 | 0.07 |  | 0.92 | 0.99 | 0.75 | 0.07 |  | 0.92 | 0.99 | 0.76 | 0.07 |
|  |  |  |  | y | 0.94 | 1.00 | 0.75 | 0.08 |  | 0.94 | 1.00 | 0.82 | 0.05 |  | 0.95 | 1.00 | 0.75 | 0.07 |
|  |  |  |  | z | 0.91 | 0.98 | 0.75 | 0.07 |  | 0.92 | 0.99 | 0.76 | 0.07 |  | 0.92 | 0.99 | 0.75 | 0.07 |
|  | MDC95 | |  | x | 0.35 | 0.70 | 0.10 | 0.16 |  | 0.36 | 0.77 | 0.15 | 0.19 |  | 0.35 | 0.77 | 0.10 | 0.18 |
|  | (mm) | |  | y | 0.22 | 0.62 | 0.02 | 0.14 |  | 0.24 | 0.53 | 0.08 | 0.13 |  | 0.23 | 0.62 | 0.02 | 0.13 |
|  |  |  |  | z | 0.32 | 0.68 | 0.09 | 0.18 |  | 0.38 | 0.71 | 0.04 | 0.21 |  | 0.35 | 0.71 | 0.04 | 0.19 |

Abs. diff, absolute difference; ICCs, intra- and inter- class correlation coefficients; MDC95, minimal detectable change with 95% confidence; Max, maximum; Min, Minimum; SD, standard deviatio

**
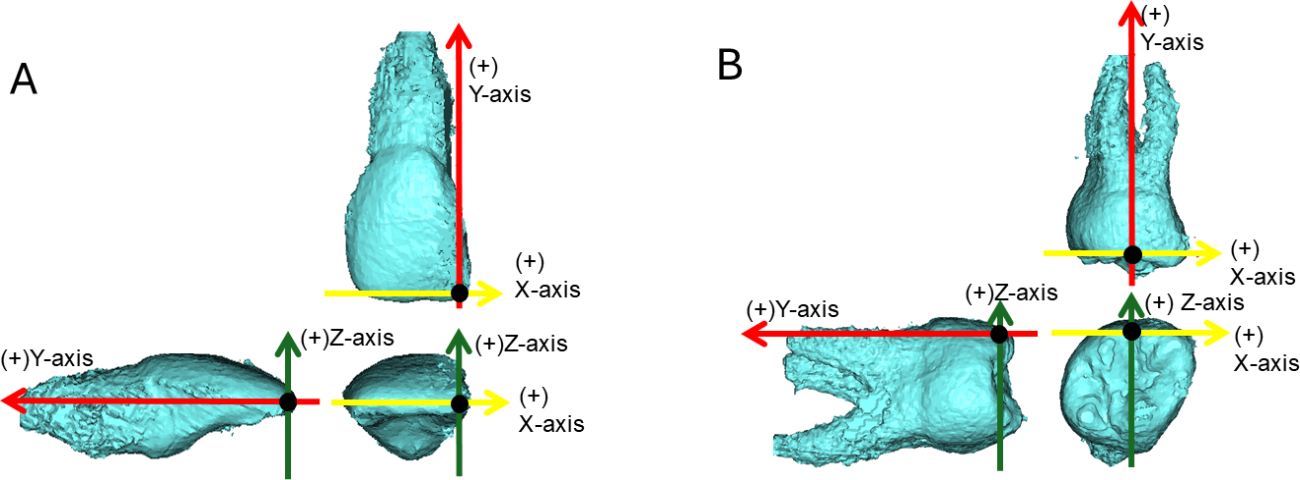
Supplementary Fig S1. Definition of coordinate axes in the maxillary right central incisor and first molar**

Definition of coordinate axes for the maxillary right central incisor (A) and maxillary right first molar (B). The origin was defined as the mesial point of the incisal edge of the central incisor and junction of the buccal groove with the occlusal surface of the first molar. The coordinate system was defined such that the X-axis corresponded to the mesiodistal direction, the Y-axis corresponded to the tooth’s axial direction, and the Z-axis corresponded to the buccolingual direction.

**
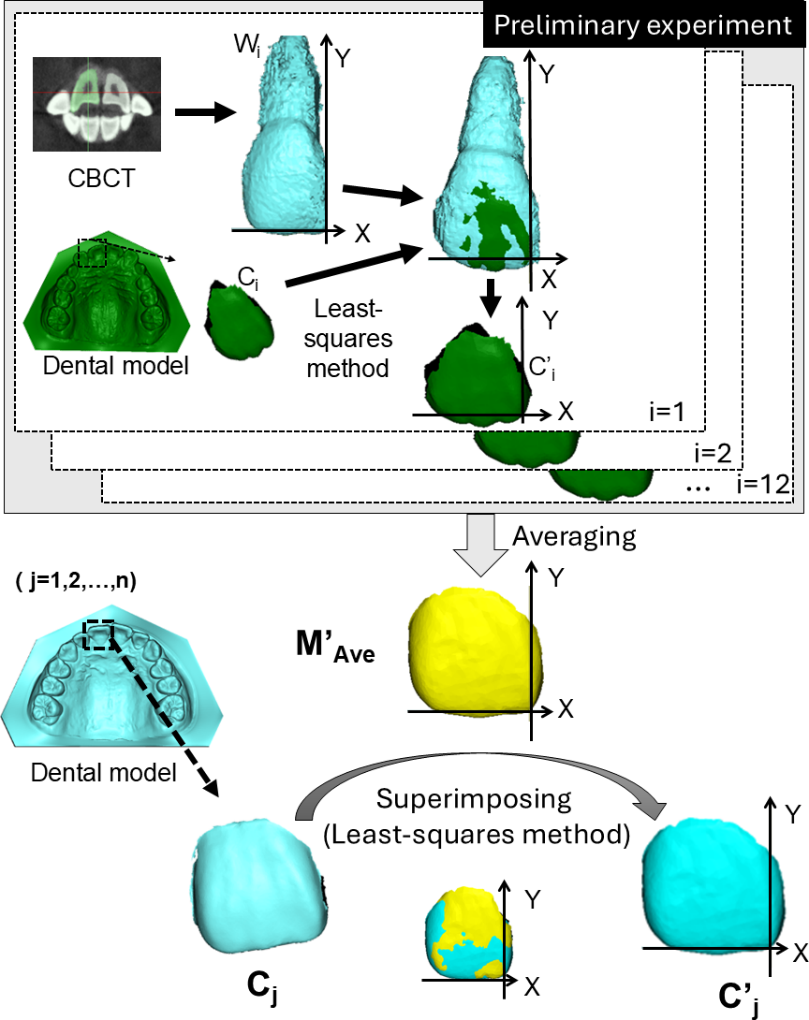
**

**Supplementary Fig S2. Schematic illustration of decision-making of standardization of the coordinate system.**

Wᵢ: 3D surface data of the tooth crown and root, extracted from CBCT and aligned with defined coordinate axes (see Supplementary Fig. S3).
Cᵢ: 3D crown surface data extracted from dental models.
C′ᵢ: Cᵢ with assigned coordinate axes.
M′ₐᵥₑ: Average crown shape model with coordinate axes.
Cⱼ: 3D crown surface data of each patient.
C′ⱼ: Cⱼ with assigned coordinate axes.
Coordinate axes for all patient crowns were established by superimposing M′ₐᵥₑ onto each Cⱼ using the least-squares method.

**
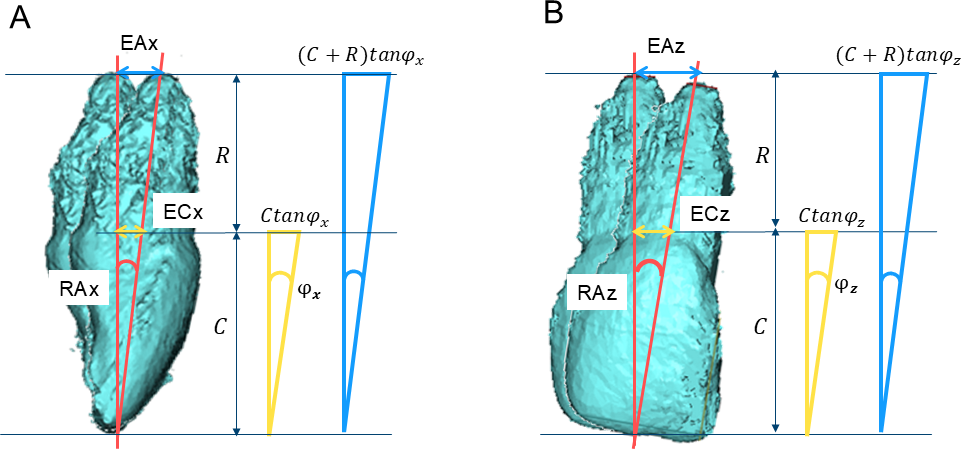
 Supplementary Fig S3. Accuracy of the new coordinate axis setting method**

RAx: Rotation angle of the tooth axis in the X-direction (φₓ, °);

ECx: Error at the cervical region in the X-direction (C·tanφₓ, mm)

ERx: Error at the root apex in the X-direction ((C + R)·tanφₓ, mm)

RAz: Rotation angle in the Z-direction (φ_z, °);

ECz: Error in the cervical region in the Z-direction (C·tanφ_z, mm)

ERz: Error at the root apex in the Z-direction ((C + R)·tanφ_z, mm).

To evaluate the accuracy of the proposed coordinate axis setting method, the rotation angles of the tooth axes were calculated by superimposing the 3D crown data with the axes set by the new method onto C′ᵢ (regarded as the true axis) using the least-squares method. Crown length (C) and root length (R) were measured parallel to the Y-axis in Wᵢ with the true coordinate system. Errors in the tooth axis orientation were estimated using the tangents of the rotation angles (φₓ and φ_z) in the X- and Z-directions, respectively.

**
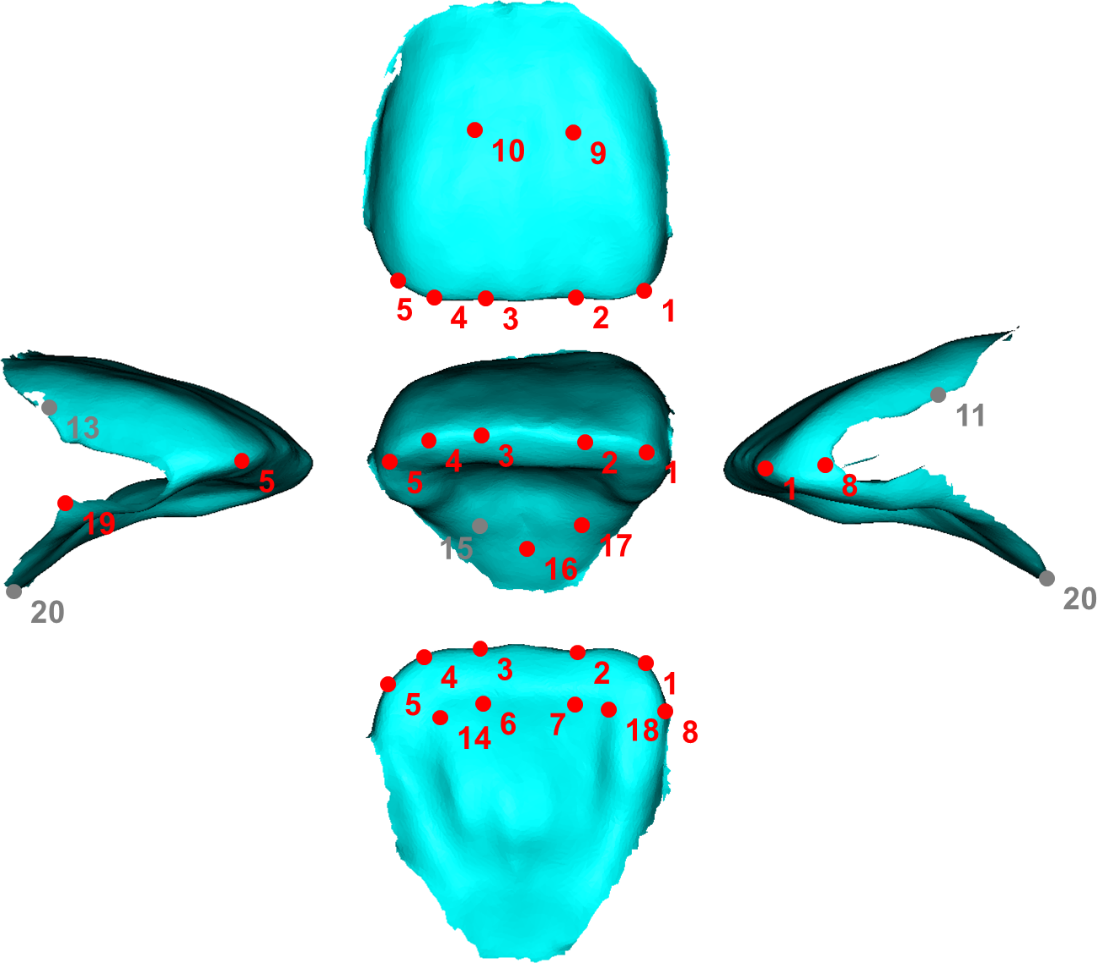
**

**Supplementary Fig S4. Twenty-one anatomical landmarks (p1, p2, …, p21) on the maxillary right central incisor.** Red denotes the anatomical landmarks adopted as reliable landmarks with ICC ≥ 0.75; gray denotes those with ICC < 0.75.

**
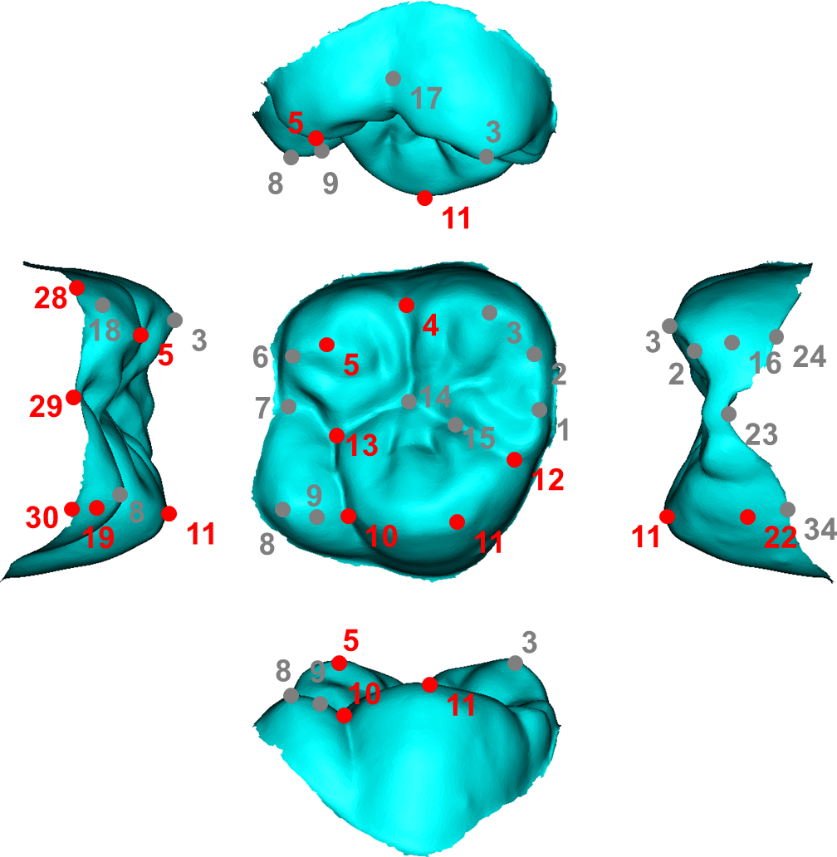
**

**Supplementary Fig S5. Thirty-four anatomical landmarks (p1, p2, …., p34) on the maxillary right first molar.** Red denotes the anatomical landmarks adopted as reliable landmarks with ICC ≥ 0.75 and gray denotes those with ICC < 0.75.

**References**

1. Smith BG, Knight JK. An index for measuring the wear of teeth. British Dental Journal 1984;156:435-438.

2. Koo TK, Li MY. A guideline of selecting and reporting intraclass correlation coefficients for reliability research. Journal of Chiropractic Medicine 2016;15:155-163.
